# Spin waves and orbital contribution to ferromagnetism in a topological metal

**DOI:** 10.1038/s41467-024-53152-1

**Published:** 2024-10-16

**Authors:** Wenliang Zhang, Teguh Citra Asmara, Yi Tseng, Junbo Li, Yimin Xiong, Yuan Wei, Tianlun Yu, Carlos William Galdino, Zhijia Zhang, Kurt Kummer, Vladimir N. Strocov, Y. Soh, Thorsten Schmitt, Gabriel Aeppli

**Affiliations:** 1 Paul Scherrer Institut, Villigen PSI, Switzerland; 2 European X-Ray Free-Electron Laser Facility GmbH, Schenefeld, Germany; 3 Anhui Province Key Laboratory of Condensed Matter Physics at Extreme Conditions, High Magnetic Field Laboratory, Chinese Academy of Sciences, Hefei, China; 4 Department of Physics, School of Physics and Optoelectronics Engineering, Anhui University, Hefei, China; 5 Hefei National Laboratory, Hefei, China; 6 European Synchrotron Radiation Facility, Grenoble, France; 7 Department of Physics, ETH Zurich, Zurich, Switzerland; 8 Quantum Center, ETH Zurich, Zurich, Switzerland; 9 Institut de Physique, EPF Lausanne, Lausanne, Switzerland

**Keywords:** Ferromagnetism, Electronic properties and materials, Magnetic properties and materials, Ferromagnetism

## Abstract

Honeycomb and kagome lattices can host propagating excitations with non-trivial topology as defined by their evolution along closed paths in momentum space. Excitations on such lattices can also be momentum-independent, and the associated flat bands are of interest due to strong interactions between heavy quasiparticles. Here, we report the discovery — using circularly polarized X-rays for the unambiguous isolation of magnetic signals — of a nearly flat spin-wave band and large (compared to elemental iron) orbital moment in the metallic ferromagnet Fe_3_Sn_2_ with compact AB-stacked kagome bilayers. As a function of out-of-plane momentum, the nearly flat optical mode and the global rotation symmetry-restoring acoustic mode are out of phase, consistent with a bilayer exchange coupling that is larger than the already large in-plane couplings. The defining units of this topological metal are therefore triangular lattices of octahedral iron clusters rather than weakly coupled kagome planes. The spin waves are strongly damped when compared to elemental iron, opening the topic of topological boson–fermion interactions for deeper exploration within this material platform.

## Abstract (web summary)

Kagome lattices host a plethora of topological phenomena. Here, the authors identify nearly flat spin-wave bands in part of the Brillouin zone and large orbital moments in the metallic kagome ferromagnet Fe_3_Sn_2_.

## Introduction

Technology has long relied on transition metal-based ferromagnets, with their high Curie temperatures and many other convenient properties, such as tunable hardness, which allows a range of applications from motors to electrical transformer cores. The basic understanding of the magnetism of such compounds needed to await the development of the quantum theory of metals in the mid-twentieth century, but there continue to be surprises concerning fundamental properties such as the voltages developed transverse to electrical currents. An example of an intermetallic compound consisting of two very common elements – iron and tin – which challenges even the contemporary quantum theory of solids is Fe_3_Sn_2_.

**Fig. 1:**
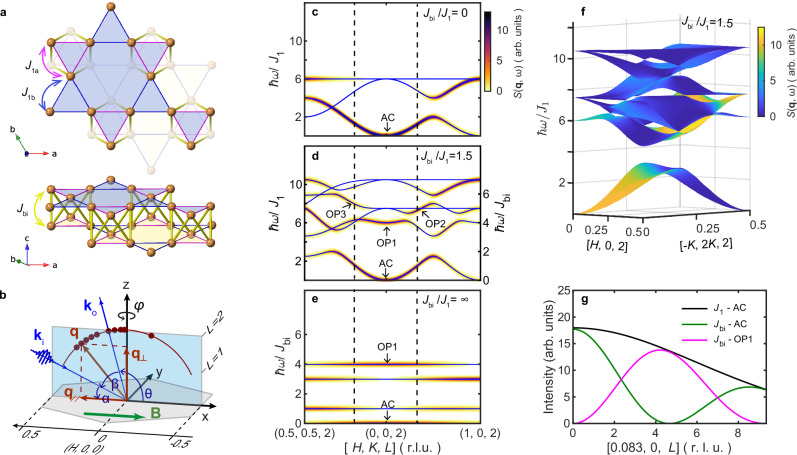
Kagome bilayer of Fe_3_Sn_2_, experimental geometry, and spin waves for an AB kagome bilayer. **a** Top (upper) and side (bottom) view of the kagome bilayer structure of Fe atoms in Fe_3_Sn_2_. The arrows illustrate the potentially major exchange couplings in the bilayer: the in-plane nearest-neighbor interaction *J*_1a_/*J*_1b_, with a (b) indicating the shorter (longer) bond in the breathing kagome lattice, and the bilayer coupling *J*_bi_. **b** Scattering geometry and sample orientation in the experiment. The gray hexagon represents the first Brillouin zone in the *L* = 0 plane, where *H, K,* and *L* are the Miller indices in reciprocal lattice units (r. l. u.). **k**_i_ and **k**_o_ are the incident and emitted X-rays, while **q** is the total momentum transfer. The green arrow indicates the applied in-plane magnetic field **B**. The arc indicates the momentum trajectory in the reciprocal space for a fixed scattering angle 180° − *β* = 130° (dark red), and the dots indicate the measured momentum points in the (*H*, 0, *L*) plane. **c**–**e** show the simulated spin-wave dispersions and spin–spin correlations (in momentum space) based on linear spin-wave theory for different *J*_bi_/*J*_1_ ratios in the *L* = 2 plane. The blue solid lines are the dispersions, and the false color intensity indicates the strength of the spin–spin correlations. The dashed lines indicate the boundaries of in-plane momentum that can be reached for a 130° fixed scattering angle. **f** 2D spin-wave dispersion and spin–spin correlations in the [*H*, *K*, 2] plane for *J*_bi_/*J*_1_ = 1.5. **g** *L* dependence of the intensities of spin-wave modes at fixed **q**_//_ = (0.083, 0): the black line indicates the acoustic (AC) mode with only *J*_1_ interaction, while the green and magenta lines indicate the acoustic (AC) mode and the bilayer split optical mode (OP1) with *J*_bi_ interaction.

Fe_3_Sn_2_ is a ferromagnet with a high Curie temperature of *T*_C_ ≈ 640 K^1–3^ and consists of kagome bilayers stacked along **c**-axis with the crystal structure belonging to the space group R-3m(1)^4^. The kagome layers are composed of two different sets of equilateral triangles with different Fe–Fe distances as indicated by the magenta and blue bonds in Fig. 1a^4^ and are stacked with an offset along the (1, −1) in-plane lattice direction. Two key questions about Fe_3_Sn_2_ follow from the initial impression, based on its high-temperature metallic ferromagnetism and the substantial Fe content, that the material is simply a diluted version of elemental iron.

The first question concerns the orbital angular momentum **L** in Fe_3_Sn_2_ that is almost entirely quenched for elemental Fe but has not been directly measured for Fe_3_Sn_2_, notwithstanding numerous thermodynamic and electrical properties which can only begin to be understood if **L** and the spin–orbit coupling (SOC) are taken into account. The most prominent is a large anomalous Hall effect^5,6^ and a transition around 120 K where the preferred magnetization direction rotates from the **c**-axis towards the kagome planes on cooling^1,2,7^. The latter has been a topic since 1970, with recent works unequivocally confirming that it is of first order and could be explained by the crossing of electronic free energies for the different magnetization directions^8,9^. Theory including the SOC as a key ingredient predicts that new phases, including states with fractionally charged quasiparticles, could emerge due to strong interactions for electrons occupying flat bands^10–12^. Recently, indications of fractionalized charge at zero magnetic field have been reported for Fe_3_Sn_2_^13^. Other discoveries center on the very large number of topologically non-trivial band crossings of Weyl character within 10 meV of the Fermi level^14,15^; for iron, there are a handful of Weyl nodes but they are further away from the Fermi level^16^. Density functional calculations show that the disposition of Weyl nodes near the Fermi level for Fe_3_Sn_2_ responds strongly to rotations of the magnetization, which accounts for the manipulation of electronic properties via modest external fields^14,17–19^.

The second question is whether there is any physical property of three-dimensional Fe_3_Sn_2_ approaching that of an ideal kagome ferromagnet with short-range interactions, including Dirac crossings and perfectly flat bands among the spin waves, which, analogous to the electronic bands, can become topologically non-trivial with additional anisotropic or antisymmetric interactions. Such topological magnons are indeed observed in a quasi-2D kagome ferromagnetic insulator – the metal-organic compound Cu[1,3-benzenedicarboxylate (bdc)]^20^. With different stackings of the kagome planes, the bands, and topology are further modified, which can lead to new phenomena^21–25^. On the experimental front, topological Dirac magnons, but no flat bands, have been reported for a room-temperature magnet YMn_6_Sn_6_^26^ consisting of ferromagnetic kagome double layers stacked with a simple vertical shift (AA-stacking).

Figure 1a illustrates that the AB bilayers in Fe_3_Sn_2_ are quite compact with the short 2.584 Å inter-planar Fe–Fe bonds (yellow) nearly the same as the shorter 2.582 Å in-plane bonds (magenta) characterizing the breathing kagome planes with the alternating longer 2.732 Å bonds (blue)^3^, yielding nearly perfect Fe octahedra. The bilayers are sandwiched between honeycomb Sn layers with a much longer inter-bilayer than intra-bilayer distance, leading to possible quasi-2D behavior of the spin degrees of freedom (DOF) on the Fe atoms. The bilayer structure suggests that the most relevant magnetic interactions in Fe_3_Sn_2_ are the in-plane nearest-neighbor interaction *J*_1a_ (*J*_1b_) in the small (large) triangles and the bilayer interaction *J*_bi_. Neglecting the difference between *J*_1a_ and *J*_1b_, i.e., setting *J*_1a_ = *J*_1b_ = *J*_1_, the spin Hamiltonian of the bilayer structure can be expressed as:

1 $${{\mathscr{H}}}={\sum}_{n=1,2}{\sum}_{\left\langle i,\, j\right\rangle }{J}_{1}{{{\bf{S}}}}_{i}^{n}\cdot {{{\bf{S}}}}_{j}^{n} \,+{\sum}_{{\left\langle i,\, j\right\rangle }_{1,2}}{J}_{{bi}}{{{\bf{S}}}}_{i}^{1}\cdot {{{\bf{S}}}}_{j}^{2}$$

In Equation (1) *n* = 1, 2 is a layer index, 〈*i*, *j*〉 labels nearest-neighbor pairs within a layer, and 〈*i*,*j*〉_1,2_ labels nearest-neighbour pairs between the two layers. The spin-wave structure is then determined by the ratio *J*_bi_/*J*_1_. Figure 1c–f show the dispersion and spin–spin correlations simulated by the spinW package^27^ for *S* = 1 with three characteristic values of *J*_bi_/*J*_1_, namely, the weak bilayer coupling limit (Fig. 1c) which reduces to the single kagome plane for *J*_bi_ = 0, the intermediate bilayer coupling regime (Fig. 1d) for *J*_bi_∼*J*_1_, and the strong bilayer coupling limit (Fig. 1e) which reduces to independent octahedral molecules of Fe arranged on a triangular (not kagome) lattice for *J*_bi_/*J*_1_ = ∞. The bilayer coupling will lift the parity degeneracy, splitting the spin-wave bands into even and odd modes with opposite out-of-plane momentum dependence^28,29^ as shown in Fig. 1g. On increasing the bilayer coupling, the spin waves show varied band crossings as a function of both momentum and *J*_bi_/*J*_1_ as the split OP1 band evolves from the lowest optical mode to the highest in the strong bilayer coupling limit. Figure 1f shows the 2D dispersion for *J*_bi_/*J*_1_ = 1.5, which has many crossing points in the optical bands. With additional anisotropic or antisymmetric interactions, gaps can be opened at these points.

To summarize, notwithstanding many experimental and theoretical papers dealing with Fe_3_Sn_2_, there are no clearly-resolved spectroscopic data confirming an impact of the kagome lattice geometry on either the charge or spin degrees of freedom. In addition, the relative orbital and spin contributions to the magnetism, as well as the underlying spin Hamiltonian, are unknown. Therefore, we have used X-ray magnetic circular dichroism (MCD) to establish the orbital component of the magnetic moment and to perform resonant inelastic scattering (RIXS) to determine the magnetic Hamiltonian. The MCD X-ray absorption spectroscopy (XAS) data reveal a much larger orbital contribution to the ferromagnetism than for elemental iron. Furthermore, we identify with MCD RIXS^30^ both conventional acoustic spin waves, with stiffness similar to that found at very low energy transfers (<2 meV) from neutron scattering from powders^31^, as well as an optical mode with at most a weak dispersion. The two spin-wave modes display out-of-phase intensities as a function of out-of-plane momentum, consistent with even and odd modes (Fig. 1g) induced by the bilayer interaction *J*_bi_. The fitting to the linear spin-wave theory suggests that *J*_bi_ $\approx$ 1.5 *J*_1_, directly implying that Fe_3_Sn_2_ is far from the weakly coupled kagome bilayer limit. Furthermore, the damping of both acoustic and optical modes are considerable at all studied momenta, indicating unusually strong interactions with electron-hole pairs.

## Results

### Spin and orbital contributions to ferromagnetism

**Fig. 2:**
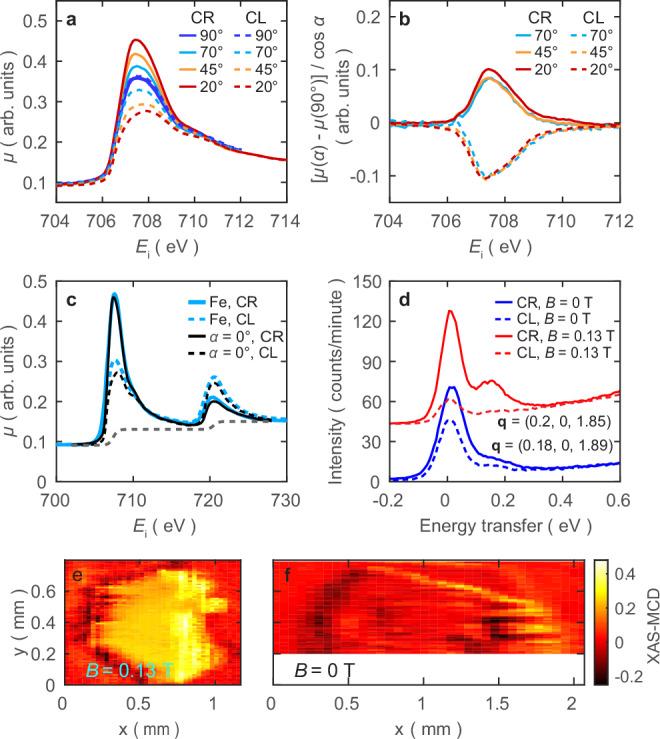
Magnetic circular dichroism of X-ray absorption spectra. **a** Absorption coefficients *µ* of Fe_3_Sn_2_ under an in-plane magnetic field (~0.13T) at different incident angles (*α*) and circular polarizations (CR and CL), extracted from X-ray absorption spectra measured by the total electron yield (TEY) method at *T* = 25 K (see Supplementary Note 1). **b** The difference between *µ* at a certain angle *α* and *µ* at *α* = 90°, scaled by a factor of $\cos \alpha$. **c** The extrapolated absorption coefficients *µ* (*α* = 0°) of Fe_3_Sn_2_ (black lines) for CR and CL polarization. The thick blue lines are the absorption coefficients of pure iron^32^. The gray dashed line is a two-step-like function for removing *L*_3_ and *L*_2_ edge jumps. The height of the step at *L*_3_ is twice the height at the *L*_2_ edge^32^. **d** RIXS spectra measured at *T* = 25 K and incident energy *E*_i_ = 707.4 eV for a sample with in-plane magnetic field (red curves, *α* = 40°) and without magnetic field (blue curves, *α* = 43°). **e** and **f** show the XAS-MCD signals ((TEY_CR_ – TEY_CL_)/(TEY_CR_ + TEY_CL_)) at Fe *L*_3_-edge resonance scanned across the sample surface at *T* = 25 K with and without magnetic field, respectively. The incident angle *α* is 20°. The scan step along x is 0.03 mm and 0.06 mm for (**e** and **f**), respectively, and 0.01 mm along y for both. The x and y directions are depicted in Fig. 1b.

Figure 1b shows the experimental geometry for XAS and RIXS measurements, where the circular polarization of the incident beam is specified while the outgoing polarizations are not resolved. To observe MCD, the magnetic domains in the sample need to be aligned, so we apply a magnetic field (~0.13 T) along the (*H*, 0, 0) direction lying in the kagome planes. Figure 2 summarizes the MCD XAS results for the Fe_3_Sn_2_ sample. The data are collected by the total electron yield (TEY) method at different incident angles *α* and photon polarizations CL (left circular) and CR (right circular) at *T* = 25 K. We extract the absorption coefficient *μ* as described in Supplementary Note 1. XAS-MCD is maximized when the helicity vector for the photon is parallel to the sample magnetization. As shown in Fig. 2a, *μ* shows no difference between CR and CL at normal incidence (*α* = 90^°^), while the difference increases with decreasing *α* from normal to grazing incidence. Figure 2b shows the normalized differences [*μ* (*α)* − *μ* (90^°^)] */* $\cos \alpha$ as a function of *α*, showing a collapse onto two curves of equal magnitude and opposite sign determined by the sign of the photon helicity. Thus, the difference simply follows the factor $\cos \alpha$, consistent with the simplest theory for the variation of XAS-MCD with the angle between the helicity of the photon and the sample magnetization **M**. Using the $\cos \alpha$ factor, we can extrapolate the XAS-MCD for *α* = 0^°^ (helicity of photon fully parallel (CR) or anti-parallel (CL) to **M**). Figure 2c displays the result together with the previous XAS-MCD result for pure iron (thick blue lines)^32^. Fe_3_Sn_2_ shows a larger dichroism at the *L*_3_-edge than pure iron. By the sum rules for XAS-MCD, the orbital and spin magnetic moments can be determined^33,34^. We assess the orbital moment per hole of an iron site to be *m*_orb_~0.13 *μ*_*B*_, and the spin moment (of the same sign) per hole to be *m*_spin_ $\cdot (1+\frac{7\left\langle {T}_{z}\right\rangle }{2\left\langle {S}_{z}\right\rangle })$ ∼ 0.6 *μ*_*B*_, where 〈*T*_z_〉 is the expectation value of the magnetic dipole operator and 〈*S*_z_〉 is equal to half of *m*_spin_ in Hartree atomic units. The assessed values are for the moment per hole at one Fe atom, so they need to be multiplied by the number of *d*-orbital holes in an iron atom to obtain the moments per Fe atom. While the spin moment *m*_spin_ in Fe_3_Sn_2_ is close to the value for pure iron, the orbital moment is much larger, with *m*_*orb*_ */m*_*spin*_~0.22 in contrast to 0.043 for pure iron^32^.

Using an XAS-MCD sample scan at the absorption maximum, we characterized the moment alignment across the whole sample with (Fig. 2e) and without (Fig. 2f) an in-plane magnetic field. The measurement is done at *α* = 20^°^. As can be seen, the magnetic field of ~0.13 T polarizes the moments of the whole sample, which shows a large domain with homogeneous XAS-MCD signals. On the other hand, the sample without a magnetic field only shows weak XAS-MCD signals in small and discrete regions due to averaging of the MCD signal over oppositely magnetized domains within the footprint (~100 μm × 5 μm) of the beam, which is large compared to the magnetic domain size^9^.

The large and homogeneous ferromagnetic domain achieved with the external magnetic field also allows the measurement of RIXS MCD for a single magnetic domain. Figure 2d shows a comparison of the RIXS spectra for samples with and without magnetic field. The former shows much more pronounced dichroism than the spectra taken without a field, with a peak around 0.15 eV almost fully suppressed for CL incident polarization. We note that the peak close to zero energy also shows a pronounced dichroism. As non-Bragg elastic scattering has no MCD in a fully polarized crystalline ferromagnet, this peak must be derived from low-energy excitations with an origin similar to that of the peak at 0.15 eV, which we identify as magnons in the analysis below.

### Spin waves in circular dichroic RIXS

**Fig. 3:**
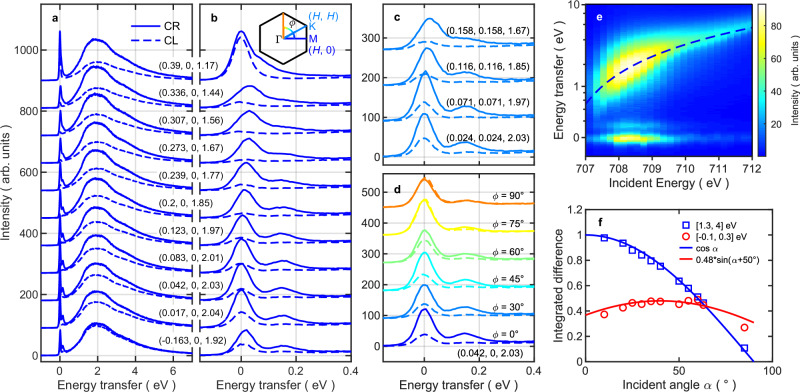
RIXS spectra with momentum and incident energy dependence. **a**–**d** RIXS spectra with CR and CL polarizations at Fe *L*_3_ resonance (*E*_i_ = 707.4 eV) and *T* = 25 K: **a** and **b** for (*H*, 0, *L*), **c** for (*H*, *H, L*), and **d** at different azimuthal angles. Here the scattering angle is fixed to 130°, so the out-of-plane momentum *L* varies as shown by the dark-red arc in Fig. 1b. The inset hexagon in **b** indicates the Brillouin zone and the lines indicate the in-plane momentum directions. **e** The RIXS intensity map as a function of energy transfer and incident energy ${hv}$ for CR polarization at *T* = 25 K; the y-axis is logarithmic, and the dashed line indicates a linear dependence on the incident energy. **f** Integrated intensities of RIXS MCD in (**a**), in an energy interval of [1.3 eV, 4 eV] (blue squares) and [−0.1 eV, 0.3 eV] (red circles) as a function of incident angle *α*. The blue line is a curve of $\cos \alpha$, and the red line is $0.48\cdot \sin (\alpha+50 ^\circ )$. The integrated intensities are divided by the size of the integrated energy interval and normalized by a scaling factor so that the fitting prefactor of the blue squares to $\cos \alpha$ is 1.

Figure 3 presents the RIXS results for a single in-plane polarized magnetic domain of Fe_3_Sn_2_ at *T* = 25 K, which are measured at 130° fixed scattering angle with a **q** trajectory shown as the dark-red arc in Fig. 1b and the incident energy tuned to the Fe *L*_3_-edge resonance at ~707 eV. The data were collected at the ADRESS beamline of Swiss Light Source (SLS)^35,36^, with an instrumental resolution of ~74 meV full-width-at-half-maximum (FWHM). The displayed spectra are corrected by the self-absorption factors with the outgoing absorption coefficients averaged among different polarizations (see Supplementary Note 2). Figure 3a shows the momentum-dependent spectra for **q** = (*H*, 0, *L*(*H*)) in a full energy transfer range, while Fig. 3b–d show the spectra for low-energy transfer for **q** = (*H*, 0, *L*(*H*)) and (*H*, *H, L’*(*H*)), and with azimuthal (φ) rotation, respectively. The RIXS spectra can be separated into two parts, a broad high-energy peak above 0.4 eV (centered around 2 eV) and low-energy peaks below 0.3 eV. To clarify the nature of these two response components, we measured an incident photon energy-dependent RIXS map at **q** = (0.123, 0, 1.97), as shown in Fig. 3e. The high-energy peak (~1 eV and larger) shifts to higher energy transfer as the incident energy *E*_i_ increases, which is characteristic for ‘fluorescence-like’^37^ behavior, while the low-energy peaks stay at fixed energy transfer. The latter Raman-like behavior suggests a collective nature of the low-energy excitations^38^. A clear RIXS-MCD effect, i.e., different intensities for CL and CR helicity of the incident X-rays, appears for both the low- and high-energy excitations. However, the momentum or incident angle dependences of the MCD for the fluorescence and Raman responses are markedly different. Figure 3f displays the integrated MCD intensities in the low- (−0.1 to 0.3 eV) and high- (1.3–4 eV) energy ranges of spectra along the (*H*, 0) direction (Fig. 3a), which are shown *vs*. the incident angle *α*. The high-energy dichroism follows a $\cos \alpha$ dependency as does the XAS-MCD, suggesting that it originates mainly from the absorption step in the RIXS process and is trivially proportional to the amount of core holes of the intermediate states created in the absorption step, while the final states are mostly irrelevant. In contrast, the low-energy dichroism depends only weakly on *α* and still shows a large dichroism even close to normal incidence, where the dichroism in XAS disappears. This suggests that the excited final states are selectively chosen by the different photon helicities and are responsible for the dichroism together with the initial states. When the sample and magnetic field (fixed along $(H,0,\,0)$ to maintain a fixed magnetic state) are rotated to lie perpendicular to the scattering plane, the entire MCD effect fades out gradually according to $\cos \phi$, as shown in Fig. 3d.

The non-trivial RIXS MCD for the low-energy excitations encodes the specific nature of the final excited states in the solid. In the approximation where core-hole excitations on different sites are uncorrelated, the RIXS cross-section can be written^39^ as a sum of local terms representing the resonances multiplied by photon polarization factors and correlation functions formed between magnetization operators at the magnetic atom sites. For spin-wave scattering, to the lowest order in the magnetization operators, this results in a (zero-temperature) cross-section proportional to

2 $$I\propto {\sum}_{{\lambda }^{{\prime} }}{\left |\langle {\lambda }^{{\prime} }| {{{\boldsymbol{\varepsilon }}}}_{{{\rm{o}}}}^{*}\times {{{\boldsymbol{\varepsilon }}}}_{{{\rm{i}}}} \cdot {\hat{{{\bf{M}}}}}_{{{\bf{q}}}} | \lambda \rangle \right|}^{2} \cdot \delta \left({E}_{\lambda }-{E}_{{\lambda }^{{\prime} }}-{{\hslash }}\omega \right )$$

Here, **ε**_i_ and **ε**_o_ are polarization vectors for incident and outgoing photons, respectively. The operator ${\hat{{{\bf{M}}}}}_{{{\bf{q}}}}$ is the Fourier transform of the local magnetization operator$\,\hat{{{\bf{M}}}}({{\bf{r}}})$, where **q** = **k**_i_–**k**_o_ is the change of the photon momentum, |λ〉 is the ferromagnetic ground state, and |λ’〉 is an excited state, which can be a magnon. The validity of expression (2) for spin waves observed by RIXS has been extensively tested^39–44^. For RIXS MCD in a Heisenberg ferromagnet, where we sum over outgoing polarizations while taking the difference between the two circular polarizations of the incident X-rays, we use the notation of Fig. 1b for a single domain sample, and obtain (see the details in the “Methods” section):

3 $${I}_{{{\rm{RIXS}}}-{{\rm{MCD}}}}\propto {\mathrm{sin}}\beta \cdot \sin \left(\alpha+\beta \right)\cdot \cos \phi \cdot {Im}({S}^{{zy}}\left({{\bf{q}}},\omega \right))$$

where *β* is the angle between the incident and outgoing photons, α and *ϕ* are the incident and azimuthal angle, respectively. ${S}^{{zy}}\left({{\bf{q}}},\omega \right)$ is the *zy* element of the dynamic spin–spin correlation function, which is imaginary. This is in contrast to neutron scattering, where the off-diagonal elements of $S\left({{\bf{q}}},\omega \right)$ cancel each other due to the dipolar polarization factors^45^. For energy loss spectra, i.e., when photons lose energy in the sample to create excitations, ${Im}({S}^{{zy}}\left({{\bf{q}}},\omega \right))$ is equal to ${S}^{{yy}}\left({{\bf{q}}},\omega \right)$ and ${S}^{{zz}}\left({{\bf{q}}},\omega \right)$. Equation (3) results in the simple angular dependence of sin*β* sin(*α* + *β*) cos*ϕ* for the RIXS MCD of spin-wave excitations of a Heisenberg ferromagnet. In Fig. 3f, where *β* is fixed at 50^°^, we show that the angle dependence of the low-energy excitations follows the sin (*α* + *β*) curve very well (see Supplementary Fig. 3 for the angular dependence of the two peaks separately), which confirms the transverse (to the magnetization) spin-wave nature of the excitations.

### Analysis of the spin waves

**Fig. 4:**
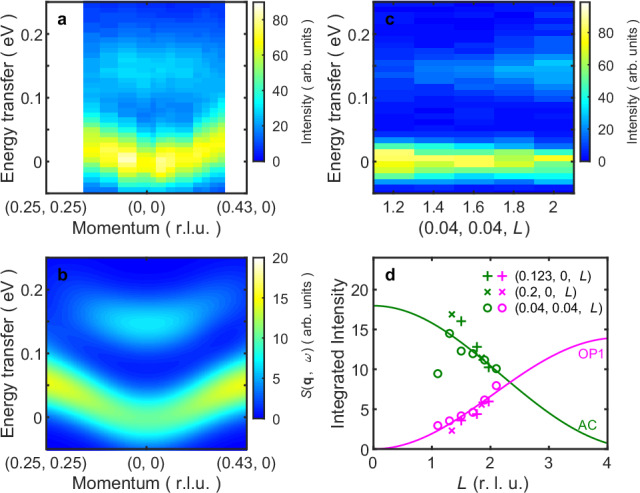
Spin excitations in q–*E* space compared to Heisenberg *J*_1_-*J*_bi_ model. **a** Intensity map of RIXS (*E*_i_ = 707.4 eV) intensity differences at *T* = 25 K between CR and CL polarizations (RIXS MCD) as a function of in-plane momentum; the data are from the fixed-scattering-angle (130°) measurements as shown in Fig. 3. The results are already corrected by polarization and self-absorption factors as described in the Supplementary Note 5. The out-of-plane momentum *L* changes as **q**_//_ changes, as shown by the red points on the red arc in Fig. 1b. **b** Spin–spin correlations ${Im}({S}^{{zy}}\left({{\boldsymbol{q}}},\omega \right)-{S}^{{yz}}\left({{\boldsymbol{q}}},\omega \right))$ simulated for a Heisenberg model with *J*_1_ = −25.0 meV and *J*_bi_ = −37.5 meV with the same **q** trajectory and resolution broadening (74 meV) as in **a**. **c** The out-of-plane *L* dependence of the RIXS MCD at fixed **q**_//_ = (0.04, 0.04), measured at ESRF with instrument resolution ~35 meV at Fe *L*_3_ resonance and *T* = 25 K. **d** Integrated intensities of the low-energy acoustic modes (AC, green symbols) and the optical modes (OP1, magenta symbols) as a function of out-of-plane momentum *L*; circles and crosses represent data from ESRF and SLS, respectively. The solid lines are the expected *L* modulations for the even and odd modes arising from the inter-plane couplings in the bilayers (bilayer interaction).

The intensity differences between CR and CL polarizations exclude phonon and elastic scatterings, isolating the magnetic contributions to the RIXS cross-section for Fe_3_Sn_2_. By correcting the intensity difference with the polarization factors of spin waves and the self-absorption effect (see Supplementary Note 5), we obtain the pure magnetic Raman signals displayed in Fig. 4a as a function of in-plane momentum **q**_//_. Here the spectra at **q** = (0.39, 0, 1.17) were excluded due to the strong contaminations on the elastic peaks as shown in Fig. 3b. There is a peak with weak momentum dependence around 0.15 eV and a low-energy acoustic mode, which moves away from zero energy as **q**_//_ increases from 0 in the quadratic manner expected for ordinary spin waves with a strong exchange interaction within the kagome planes.

To understand the contribution of inter-layer couplings, we measured the out-of-plane momentum (*L*) dependence of the RIXS MCD spectra. Figure 4c shows the *L*-dependent low-energy RIXS MCD at a fixed in-plane **q**_//_ = (0.04, 0.04), measured at the ID32 beamline of European Synchrotron Radiation Facility (ESRF), with a resolution ~35 meV FWHM. It clearly illustrates the out-of-phase *L* dependence of the two modes, exactly as expected for the even and odd modes induced by the bilayer interaction. The even and odd modes represent the in-phase and out-of-phase movements of the spins in the two planes forming the bilayer, and their intensities have *L* modulations depending on the thickness of the bilayer ${z}_{{bi}}$ with forms ${{f}^{2}({{\bf{q}}})\cdot \cos }^{2}(\pi {z}_{{bi}}L)$ and ${f}^{2}({{\bf{q}}})\cdot {\sin }^{2}(\pi {z}_{{bi}}L)$, respectively, where $f({{\bf{q}}})$ is the Fe form-factor^28,29^, as shown in Fig. 1g. In Fig. 4d, we compare the integrated intensities of the observed two peaks to these simple *L* modulations (solid lines). The circles are from the ESRF data shown in Fig. 4c at **q**_//_ = (0.04, 0.04) integrated over an interval of [−0.07, 0.07] eV (green circles) and [0.07, 0.3] eV (magenta circles), while the plus and cross symbols are from the measurements at the ADRESS beamline of the SLS at **q**_//_ = (0123, 0) and **q**_//_ = (0.2, 0) shown in Supplementary Fig. 5. The observed intensities follow the *L* modulations very well, except one AC point (green circle) at *L* = 1.1, which drops off the trend due to the degradation of the sample surface at extended times after cleaving, weakening the dichroism of the acoustic mode (see “Methods” section and Supplementary Note 6 for the detailed RIXS spectra).

**Fig. 5:**
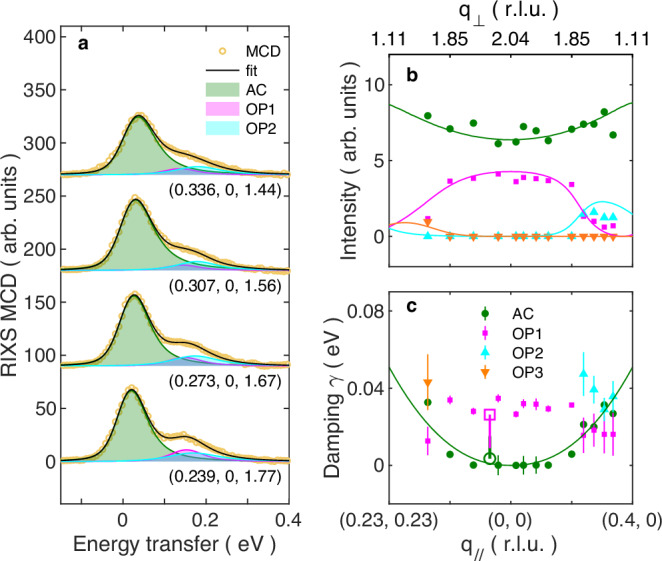
Spin-wave theory description of the RIXS magnetic circular dichroism for *T* = 25 K and *E*_i_ = 707.4 eV. **a** Examples of fitting by *J*_1_-*J*_bi_ model with damped harmonic oscillator profiles on the RIXS MCD at four relatively large **q**_//_ along (*H*, 0). The dispersion derives from *J*_1_ and *J*_bi_ being fixed as described in the text while the amplitude and damping are allowed to vary in the fits. The complete fitting for all momenta is presented in Supplementary Fig. 6. **b** The integrated intensities of the fitted spin-wave modes; the green circles and magenta squares indicate the even acoustic mode (AC) and odd optical mode (OP1), respectively. The cyan and orange triangles indicate the other optical modes that emerge at relatively large **q**_//_ along (*H*, 0) and (*H*, *H*), respectively, as shown in Fig. 1d. The solid lines indicate the simulated intensities of the spin-wave modes based on the *J*_1_ − *J*_bi_ model. **c** The fitted damping factors for the four spin-wave modes. The thin bars represent the standard errors from the fitting procedure. The green solid line corresponds to *P* | **q**_//_|^*δ*^ with *δ* = 2.26, *P* = 0.40 eV, and |**q**_//_| in reciprocal lattice units defined such that (1, 0) occurs at 1.365 Å^−1^. The open circles and squares are the fitted damping factors of AC and OP1 modes of *L*-dependent data from ESRF at **q** = (0.04, 0.04, 2.1), respectively. The thick pink bar indicates the range of fitted damping factors for the *L*-dependent data at **q** = (0.04, 0.04, *L*) with *L* varying from 2.1 to 1.1; detailed values are shown in Supplementary Fig. 4e.

To determine the magnetic interactions and spin-wave decay rates, we fit the RIXS MCD spectra shown in Fig. 4a to a convolution of a 74 meV FWHM resolution function with the sum of damped harmonic oscillator profiles:

4 $$S\left(\omega \right)=\left(n\left(\omega \right)+1\right)\cdot \frac{A\gamma \omega }{{\left({\omega }^{2}-{\omega }_{0}^{2}\right)}^{2}+4{\gamma }^{2}{\omega }^{2}}$$

where eigenfrequencies are calculated using the spinW package^27^ applied to the bilayer Heisenberg model of Eq. (1). Here, the fitting parameters are *J*_1_, *J*_bi_, the amplitudes, and the damping factors for the relevant spin-wave modes (AC, OP1, OP2, and OP3 in Fig. 1d), which show non-zero intensities in the accessed momentum space. The values of *J*_1_ and *J*_bi_ determine the dispersions, which we set to be *ω*_0 _in the fitting function (4). As the OP2 and OP3 modes have negligible intensities at small **q**_//_ (Fig. 1d), we manually set them to zero for **q**_//_ ≤ (0.2, 0) along (*H*, 0) and **q**_//_ ≤ (0.12, 0.12) along (*H*, *H*) to improve convergence. The fitting applies to all the spectra shown in the intensity map of Fig. 4a and gives the best results for *J*_1_ = −25.0 (±2.9) meV and *J*_bi_ = −37.5 (±1.8) meV. *J*_bi_ is mostly determined by the minimal energy of the optical OP1 mode, i.e., the energy splitting between the even and odd modes, while *J*_1_ is mostly determined by the stiffness of the acoustic mode. The errors are estimated by assuming that the error in elastic peak positions (zero energy transfer) is ~10% of the FWHM of the resolution (~7 meV). We plot in Fig. 4b, the simulated spin–spin correlations ${Im}({S}^{{zy}}\left({{\boldsymbol{q}}},\omega \right)-{S}^{{yz}}\left({{\boldsymbol{q}}},\omega \right))$ for a Heisenberg model with the obtained *J*_1_ and *J*_bi_ but with damping *γ* = 0. The **q** trajectory is the same as in the experiment and the spectra are broadened by a Gaussian with FWHM = 74 meV. The results look very similar to the data in Fig. 4a. Nonetheless, the peak intensity of the acoustic mode does not seem to grow along the q trajectory in the experiments (Fig. 4a) as it does in the simulation (Fig. 4b). This apparent discrepancy is a result of the increased damping factor of the acoustic mode in the experiments for larger *q* values, which will be explained by the fitting results in Fig. 5.

Figure 5a shows the fitted spectra at four relatively large **q**_//_ along (*H*, 0), where the hardening of the acoustic mode and emergence of the OP2 mode, whose intensity gradually exceeds that of the OP1 mode with increasing **q**_//,_ yield an excellent (but almost needless to say, not mathematically unique) account of the entire lineshape; attempts to fit these higher momentum transfer data with only two damped harmonic oscillators were not as successful. Figure 5b, c show the integrated intensities and the damping factors of the fitted spin-wave modes, respectively. The damping factor of the acoustic mode increases greatly at larger **q**_//_ values, which reduces the peak intensity and leads to the differing trends in the intensity maps of Fig. 4a, b noted above. Nevertheless, the integrated intensities follow the calculated amplitudes (solid lines) for the *J*_1_-*J*_bi_ model very well, which confirms that the adopted model is appropriate for our results.

Although *J*_1_ and *J*_bi_ may be the most important interactions, other parameters such as the inter-bilayer interaction *J*_c_, and a difference between *J*_1a_ and *J*_1b_ can exist and modify the spin waves. Spin-space anisotropy and/or non-zero *J*_c_ would be necessary for the magnetic ordering at the high Curie temperature of 640 K^46,47^, since there is no order for a pure 2D Heisenberg magnet. *J*_c_ will further introduce dispersion along *L*. However, the *L* dependence (Fig. 4c and Supplementary Fig. 4) does not reveal dispersion, which suggests that *J*_c_ is much smaller than *J*_1_ and *J*_bi_. Based both on our measurements concerning the out-of-plane momentum dependence as well as the knowledge of the small anisotropy fields^9^, the mean-field formula for the Curie temperature should provide an upper bound^46^, which, when the extended fluctuation regime in 2D systems ending in a quasi-Kosterlitz–Thouless transition is considered, can actually be reduced by approximately a factor of two^48^. The outcome of all of our (*H*, *K*, *L*)-dependent measurements is, if we set *J*_c_ = 0, ${T}_{{CMF}}=\frac{S\left(S+1\right)}{3{k}_{B}}\left({z}_{1}{ \, J}_{1}+{z}_{{bi}}{ \, J}_{{bi}}+{z}_{c}{J}_{c}\right)$ ~1360 K (where *z* is the coordination number with *z*_1_ = 4 and *z*_bi_ = 2), implying an estimated *T*_C_ of 680 K, which is remarkably close to the 640 K observed experimentally. Different *J*_1a_ and *J*_1b_ exert much subtler influence, mostly on the flatness of the optical modes and their crossing points.

### Spin-wave topology and interactions with electron-hole pairs

The Fe_3_Sn_2_ lattice is such that the bonds between Fe atoms do not see an inversion symmetric environment, implying, together with the substantial orbital angular momentum associated with the iron atoms, a non-zero Dzyaloshinskii–Moriya interaction (DMI). The DMI can open gaps at the crossing points (Fig. 1f), which leads to the question of whether the magnons will become topologically non-trivial. The answer is that the magnon flat band must be topological in the limit of single layers^49^ (i.e., where the bilayer coupling is weak), as it also is for Cu(1,3-bdc)^20^. In the opposite limit where the bilayer coupling is very strong, we have a triangular lattice with flat modes characteristic of excitations within the octahedral Fe “molecules”, and we expect trivial topology. There should therefore be a phase transition between the kagome and triangular regimes as a function of the ratio between the two couplings. The associated effects are beyond the current resolution and momentum transfer range but will be interesting to verify with further RIXS and neutron scattering studies.

Another outcome of the fitting is the exceptionally high spin-wave damping shown in Fig. 5c when compared to “local moment” ferromagnets including classic insulators such as EuO^50^ and the manganites^51^. In Heisenberg ferromagnets, the spin-wave damping can occur due to a diffusion-like process which varies in proportion to *q*^4^ for the acoustic mode^52,53^. The solid line in Fig. 5c displays the fitting of the damping of the acoustic mode by *P* | **q**_//_|^*δ*^, where |**q**_//_| is expressed in dimensionless reciprocal lattice units. The fitted result $\delta=2.26\pm 0.60$ is smaller than 4, while $P=0.40\pm 0.30$ eV is much larger than not only the small thermal energy ${k}_{B}T=0.002$ eV, but also the observed spin-wave energy of the OP1 mode. The damping of the OP1 mode (measured at SLS) is almost momentum-independent with $\gamma \approx$ 0.027 eV, corresponding to a FWHM of 0.055 eV. To account for the strong damping, electronic excitations outside the manifold of spin-wave excitations are required. An important origin of the damping could be the decay into electron-hole pairs, similar to the high damping in iron and nickel following from spin-conserving (i.e., without appealing to spin–orbit effects) decays into electron-hole pairs^54–57^. In Fe_3_Sn_2_, the electronic states and the Fermi surface respond strongly to the magnetic field and the magnetization direction^13,14,17,19^. Therefore, the electronic states can be distorted as the spin waves propagate, which in turn damps the spin waves^58^. Furthermore, special regions of the Fermi surface, such as those near band crossings, are lifted by the SOC and depend strongly on the magnetization direction^58^. Indeed, multiple Weyl nodes that are switchable by magnetization are suggested to exist close to the Fermi level^14^. Spin waves can therefore redistribute Weyl fermions thus shortening the lifetime of both. Despite the strong damping, the high-energy optical spin waves in Fe_3_Sn_2_ are still well-defined, in contrast to other related kagome magnetic metals such as FeSn^59,60^, CoSn^59^, and Co_3_Sn_2_S_2_^61–63^, where the high-energy spin waves are much less visible if at all.

## Discussion

We have exploited modern synchrotron-based X-ray technology to examine the magnetic order and excitations in the much-celebrated metallic kagome ferromagnet Fe_3_Sn_2_, allowing direct comparison to both insulating kagome ferromagnets and metallic iron. The magnetic circular dichroism of the X-ray absorption reveals that the orbital contribution to the magnetic moment is five times larger than in elemental iron where it is understood to be almost entirely quenched on account of the crystal field energies being larger than the spin–orbit interaction. This is a quantitative manifestation of the large SOC which also makes Fe_3_Sn_2_ a topological material, with numerous Weyl nodes^14^, and indeed suggests a method to calibrate the SOC strength introduced “by hand” into DFT: SOC is simply varied to obtain the measured ratio of orbital to spin contributions to the magnetization.

Furthermore, taking advantage of the magnetic circular dichroism of RIXS, we discovered two spin-wave bands that are ascribed to the even and odd modes, derived from strong bilayer coupling, by measurements of the out-of-plane momentum dependence. This means that the underlying magnetic and concomitant electronic Hamiltonians for Fe_3_Sn_2_ are remote from the limit of weakly coupled single kagome layers, thus accounting for the difficulty of finding in both computation (DFT) and experiment (angle-resolved photoelectron emission spectroscopy) the flat bands and resolved Dirac points associated with single kagome layers. Another picture emerges from these results, namely that the fundamental low-energy electronic building blocks are triangular lattices of octahedral Fe “molecules”, without the possibility for perfectly flat modes in the planar reciprocal space but with many new touching points between the greater number of modes introduced by the “molecules”. Our work thus motivates the control of these touching points by environmental and chemical parameters, as well as the theory of their topological nature. Finally, that there is a strong mixing of the optical modes with the electron-hole pair continuum is clear from their considerable damping even for $q\longrightarrow 0.$ The mixing may be due to attempted rearrangements of the Fe_3_Sn_2_ Weyl nodes caused by transient magnetization rotations associated with the spin waves. We look forward to more experiments and theories on what happens when topological electrons mix with topological bosons.

## Methods

### Sample synthesis

Single crystals of Fe_3_Sn_2_ were synthesized by a chemical vapor transport method. The stoichiometric mixture of high-purity iron powder and tin powder were sealed into an evacuated quartz tube. The mixture was heated at 800 °C for 7 days and then quenched in icy water. The sintered Fe_3_Sn_2_ was thoroughly ground and then sealed in a quartz tube (1 cm in diameter and 16 cm in length) with I_2_ (about 4 mg/cm^3^) under vacuum. The tube was put under a temperature gradient of 650 °C (source) and 720 °C (sink) for two weeks for the crystals to grow.

### Experiments

The XAS experiments, RIXS experiments with 130° fixed scattering angle, and *L*-dependent RIXS experiments at **q** = (0.123, 0, *L*) and **q** = (0.2, 0, *L*) were carried out at the ADRESS beamline of the Swiss Light Source (SLS) at the Paul Scherrer Institut^35,36^, while the *L*-dependent RIXS measurements at **q** = (0.04, 0.04, *L*) were done at the ID32 beamline of European Synchrotron Radiation Facility (ESRF). The momentum transfer **q** is denoted in reciprocal lattice units (r. l. u.), with lattice constants a = b = 5.315 Å and c = 19.703 Å. For the measurements at ADRESS, the plate-shaped crystal was cleaved in ultra-high vacuum (~2 × 10^−10^ mbar) at *T* = 25 K to yield a clean and flat surface parallel to the *a-b* plane. The scattering plane, spanned by the incident (**k**_i_) and emitted (**k**_o_) photon wave vectors, is perpendicular to the sample *a*–*b* plane, and intersects with it for **q** = (*H*, 0, 0) when $\phi=0$, as shown in Fig. 1b. The instrumental resolution of the RIXS measurements at the ADRESS beamline is ~74 meV full-width-at-half-maximum (FWHM) for 130° fixed-scattering-angle measurements, while it is ~80 meV FWHM for *L*-dependent RIXS measurements at 110° and 90° scattering angles. A magnetic field (**B**~0.13 T) along the (*H*, 0, 0) direction from a pair of permanent magnets polarized the ferromagnetic sample. At ID32 of ESRF, the crystal was cleaved in the transfer chamber (~1 × 10^−8^ mbar) at room temperature and transferred immediately into the measuring chamber and cooled down to 25 K with a vacuum ~1 × 10^−9^ mbar; the instrumental resolution is ~35 meV FWHM and the magnetic field (~0.2 T) is applied along the (*H*, *H, 0*) direction. Right (CR) and left (CL) circular polarized incident photons are employed for the measurements, while the polarizations of the emitted photons in RIXS are not resolved. All data were collected at a base temperature of ∼25 K.

### Calculation of polarization factor

Letting ${{\bf{P}}}={{{\boldsymbol{\varepsilon }}}}_{{{\rm{o}}}}^{*}\times {{{\boldsymbol{\varepsilon }}}}_{{{\rm{i}}}}$, Eq. (2) can be rewritten as:

5 $$I\propto {\sum}_{{ab}}{P}_{a}^{*}{P}_{b}{\sum}_{{\lambda }^{{\prime} }}\langle \lambda | {\hat{{{\bf{M}}}}}_{{{\bf{q}}}{{,}}a}^{{\dagger} } | {\lambda }^{{\prime} }\rangle \langle {\lambda }^{{\prime} } | {\hat{{{\bf{M}}}}}_{{{\bf{q}}}{{,}}b} | \lambda \rangle \cdot \delta ({E}_{\lambda }-{E}_{{\lambda }^{{\prime} }}-{{\hslash }}\omega )$$

where $a$ and $b$ stand for *x, y,* and *z*, which are the indices of the vector elements. The formula is very similar to the magnetic scattering of neutrons^45^, with only different polarization factors ${P}_{a}^{*}{P}_{b}$. As for neutron scattering, the second summation in the above formula is proportional to the spin–spin correlation function ${S}^{{ab}}\left({{\bf{q}}},\omega \right)$. In the linear approximation for a local moment ferromagnetic system, given that the spins are polarized in the *x* direction shown by Fig. 1b, only the elements ${S}^{{aa}}$, ${S}^{{yz}}$, and ${S}^{{zy}}$ are non-zero. While ${S}^{{xx}}$ contributes to the elastic scattering, ${S}^{{yy}}$, ${S}^{{zz}}$, ${S}^{{yz}}$ and ${S}^{{zy}}$ are related to the dynamic part, with ${S}^{{yy}}={S}^{{zz}}$ and ${S}^{{yz}}={-S}^{{zy}}$. In the scattering geometry of Fig. 1b, we can define the polarization vectors **ε**_i_ and **ε**_o_. For example, the CL and CR incident polarizations are $(-i\sin \alpha,\,1,\,-i\cos \alpha )/\sqrt{2}$ and $(i\sin \alpha,\,1,{i}\cos \alpha )/\sqrt{2}$, respectively. By summing over the possible outgoing polarizations **ε**_o_, we obtain the cross-section proportional to:

6 $$\begin{array}{c}{I}_{{CL}/{CR}}\propto \left[{(\sin \alpha )}^{2}+{\left(\sin \left(\alpha+\beta \right)\right)}^{2}\right]\cdot {S}^{{zz}}\left({{\bf{q}}},\omega \right)+{(\sin \beta )}^{2}\cdot {S}^{{yy}}\left({{\bf{q}}},\omega \right)\\ (-/+)\sin \beta \cdot \sin \left(\alpha+\beta \right)\cdot i{S}^{{yz}}\left({{\bf{q}}},\omega \right)(+/-)\sin \beta \cdot \sin \left(\alpha+\beta \right)\cdot i{S}^{{zy}}\left({{\bf{q}}},\omega \right)\end{array}$$

For energy loss as probed in RIXS, ${S}^{{zy}}$ is imaginary and equal to $i{S}^{{yy}}$. If we further include the azimuthal rotation, a $\cos \phi$ factor is needed, which altogether results in the RIXS MCD formula as Eq. (3).

## Supplementary information

Supplementary Information

Peer Review File

## Source data

Source Data

## Data Availability

The data that support the findings of this study are shown in the main text figures and the supplementary data figures. The source data generated during and/or analyzed in the current study has been deposited in the Zenodo database under the accession code 10.5281/zenodo.12721667. The original data collected at ESRF are available under the accession code 10.15151/ESRF-DC-1823716263. Source data are provided with this paper.

## References

[1] Trumpy, G., Both, E., Djéga-Mariadassou, C. & Lecocq, P. Mössbauer-effect studies of iron-tin alloys. *Phys. Rev. B* **2**, 3477–3490 (1970).

[2] Le Caer, G., Malaman, B. & Roques, B. Mössbauer effect study of Fe_3_Sn_2_. *J. Phys. F. Met. Phys.* **8**, 323–336 (1978).

[3] Fenner, L. A., Dee, A. A. & Wills, A. S. Non-collinearity and spin frustration in the itinerant kagome ferromagnet Fe_3_Sn_2_. *J. Phys. Condens. Matter* **21**, 452202 (2009). 21694002 10.1088/0953-8984/21/45/452202

[4] Malaman, B., Roques, B., Courtois, A. & Protas, J. Structure cristalline du stannure de fer Fe_3_Sn_2_. *Acta Crystallogr. Sect. B* **32**, 1348–1351 (1976).

[5] Kida, T. et al. The giant anomalous Hall effect in the ferromagnet Fe_3_Sn_2_ - a frustrated kagome metal. *J. Phys. Condens. Matter* **23**, 112205 (2011). 21358031 10.1088/0953-8984/23/11/112205

[6] Wang, Q., Sun, S., Zhang, X., Pang, F. & Lei, H. Anomalous Hall effect in a ferromagnetic Fe_3_Sn_2_ single crystal with a geometrically frustrated Fe bilayer kagome lattice. *Phys. Rev. B* **94**, 075135 (2016).

[7] Malaman, B., Fruchart, D. & Le Caer, G. Magnetic properties of Fe_3_Sn_2_. II. Neutron diffraction study (and Mössbauer effect). *J. Phys. F. Met. Phys.* **8**, 2389–2399 (1978).

[8] Kumar, N., Soh, Y., Wang, Y. & Xiong, Y. Magnetotransport as a diagnostic of spin reorientation: kagome ferromagnet as a case study. *Phys. Rev. B* **100**, 214420 (2019).

[9] Heritage, K. et al. Images of a first-order spin-reorientation phase transition in a metallic kagome ferromagnet. *Adv. Funct. Mater.* **30**, 1909163 (2020).

[10] Tang, E., Mei, J. W. & Wen, X. G. High-temperature fractional quantum Hall states. *Phys. Rev. Lett.* **106**, 236802 (2011). 21770532 10.1103/PhysRevLett.106.236802

[11] Sun, K., Gu, Z., Katsura, H. & Das Sarma, S. Nearly flatbands with nontrivial topology. *Phys. Rev. Lett.* **106**, 236803 (2011). 21770533 10.1103/PhysRevLett.106.236803

[12] Neupert, T., Santos, L., Chamon, C. & Mudry, C. Fractional quantum Hall states at zero magnetic field. *Phys. Rev. Lett.* **106**, 236804 (2011). 21770534 10.1103/PhysRevLett.106.236804

[13] Ekahana, S. A. et al. Anomalous electrons in a metallic kagome ferromagnet. *Nature* **627**, 67–72 (2024). 38448698 10.1038/s41586-024-07085-w PMC10917658

[14] Yao, M. et al. Switchable Weyl nodes in topological kagome ferromagnet Fe_3_Sn_2_. Preprint at http://arxiv.org/abs/1810.01514 (2018).

[15] Tanaka, H. et al. Three-dimensional electronic structure in ferromagnetic Fe_3_Sn_2_ with breathing kagome bilayers. *Phys. Rev. B* **101**, 161114 (2020).

[16] Gosálbez-Martínez, D., Souza, I. & Vanderbilt, D. Chiral degeneracies and Fermi-surface Chern numbers in bcc Fe. *Phys. Rev. B* **92**, 085138 (2015).

[17] Kumar, N., Soh, Y., Wang, Y., Li, J. & Xiong, Y. Tuning the electronic band structure in a kagome ferromagnetic metal via magnetization. *Phys. Rev. B* **106**, 045120 (2022).

[18] Yin, J. et al. Giant and anisotropic many-body spin–orbit tunability in a strongly correlated kagome magnet. *Nature* **562**, 91–95 (2018). 30209398 10.1038/s41586-018-0502-7

[19] Kumar, N., Soh, Y., Wang, Y., Li, J. & Xiong, Y. Anomalous planar Hall effect in a kagome ferromagnet. Preprint at http://arxiv.org/abs/2005.14237 (2020).

[20] Chisnell, R. et al. Topological magnon bands in a kagome lattice ferromagnet. *Phys. Rev. Lett.* **115**, 147201 (2015). 26551820 10.1103/PhysRevLett.115.147201

[21] Crasto De Lima, F., Miwa, R. H. & Suárez Morell, E. Double flat bands in kagome twisted bilayers. *Phys. Rev. B* **100**, 155421 (2019).

[22] Wu, F., Lovorn, T., Tutuc, E., Martin, I. & Macdonald, A. H. Topological insulators in twisted transition metal dichalcogenide homobilayers. *Phys. Rev. Lett.* **122**, 086402 (2019). 30932597 10.1103/PhysRevLett.122.086402

[23] Zelenskiy, A., Plumer, M. L., Southern, B. W., Zhitomirsky, M. E. & Monchesky, T. L. Chiral nematic and fluctuation-induced first-order phase transitions in AB-stacked kagome bilayers. *Phys. Rev. B* **108**, L060402 (2023).

[24] Thomasen, A., Penc, K., Shannon, N. & Romhányi, J. Fragility of Z2 topological invariant characterizing triplet excitations in a bilayer kagome magnet. *Phys. Rev. B* **104**, 104412 (2021).

[25] Shi, M. et al. A new class of bilayer kagome lattice compounds with Dirac nodal lines and pressure-induced superconductivity. *Nat. Commun.* **13**, 2773 (2022). 35589799 10.1038/s41467-022-30442-0 PMC9120444

[26] Zhang, H. et al. Topological magnon bands in a room-temperature kagome magnet. *Phys. Rev. B* **101**, 100405 (2020).

[27] Toth, S. & Lake, B. Linear spin wave theory for single-Q incommensurate magnetic structures. *J. Phys. Condens. Matter* **27**, 166002 (2015). 25817594 10.1088/0953-8984/27/16/166002

[28] Reznik, D., Bourges, P. & Fong, H. Direct observation of optical magnons in YBa_2_Cu_3_O_6.2_. *Phys. Rev. B* **53**, R14741–R14744 (1996). 10.1103/physrevb.53.r14741 9983344

[29] Xie, T. et al. Odd and even modes of neutron spin resonance in the bilayer iron-based superconductor CaKFe_4_As_4_. *Phys. Rev. Lett.* **120**, 267003 (2018). 30004765 10.1103/PhysRevLett.120.267003

[30] Elnaggar, H. et al. Magnetic contrast at spin-flip excitations: an advanced X-ray spectroscopy tool to study magnetic-ordering. *ACS Appl. Mater. Interfaces* **11**, 36213–36220 (2019). 31495171 10.1021/acsami.9b10196 PMC6778912

[31] Dally, R. L., Phelan, D., Bishop, N., Ghimire, N. J. & Lynn, J. W. Isotropic nature of the metallic kagome ferromagnet Fe_3_Sn_2_ at high temperatures. *Crystals* **11**, 307 (2021). 10.3390/cryst11030307 PMC10938373 38487672

[32] Chen, C. T. et al. Experimental confirmation of the X-ray magnetic circular dichroism sum rules for iron and cobalt. *Phys. Rev. Lett.* **75**, 152–155 (1995). 10059138 10.1103/PhysRevLett.75.152

[33] Thole, B. T., Carra, P., Sette, F. & Van Der Laan, G. X-ray circular dichroism as a probe of orbital magnetization. *Phys. Rev. Lett.* **68**, 1943–1946 (1992). 10045260 10.1103/PhysRevLett.68.1943

[34] Carra, P., Thole, B. T., Altarelli, M. & Wang, X. X-ray circular dichroism and local magnetic fields. *Phys. Rev. Lett.* **70**, 694–697 (1993). 10054179 10.1103/PhysRevLett.70.694

[35] Ghiringhelli, G. et al. SAXES, a high resolution spectrometer for resonant x-ray emission in the 400-1600 eV energy range. *Rev. Sci. Instrum.* **77**, 113108 (2006).

[36] Strocov, V. N. et al. High-resolution soft X-ray beamline ADRESS at the Swiss Light Source for resonant inelastic X-ray scattering and angle-resolved photoelectron spectroscopies. *J. Synchrotron Radiat.* **17**, 631–643 (2010). 20724785 10.1107/S0909049510019862 PMC2927903

[37] Bisogni, V. et al. Ground-state oxygen holes and the metal-insulator transition in the negative charge-transfer rare-earth nickelates. *Nat. Commun.* **7**, 13017 (2016). 27725665 10.1038/ncomms13017 PMC5062575

[38] Minola, M. et al. Collective nature of spin excitations in superconducting cuprates probed by resonant inelastic X-ray scattering. *Phys. Rev. Lett.* **114**, 217003 (2015). 26066453 10.1103/PhysRevLett.114.217003

[39] Haverkort, M. W. Theory of resonant inelastic X-ray scattering by collective magnetic excitations. *Phys. Rev. Lett.* **105**, 167404 (2010). 21231013 10.1103/PhysRevLett.105.167404

[40] Ament, L. J. P., Van Veenendaal, M., Devereaux, T. P., Hill, J. P. & Van Den Brink, J. Resonant inelastic X-ray scattering studies of elementary excitations. *Rev. Mod. Phys.* **83**, 705–767 (2011).

[41] Braicovich, L. et al. Magnetic excitations and phase separation in the underdoped La_2-x_Sr_x_CuO_4_ superconductor measured by resonant inelastic X-ray scattering. *Phys. Rev. Lett.* **104**, 077002 (2010). 20366909 10.1103/PhysRevLett.104.077002

[42] Guarise, M. et al. Measurement of magnetic excitations in the two-dimensional antiferromagnetic Sr_2_CuO_2_Cl_2_ insulator using resonant X-Ray scattering: Evidence for extended interactions. *Phys. Rev. Lett.* **105**, 157006 (2010). 21230933 10.1103/PhysRevLett.105.157006

[43] Schlappa, J. et al. Spin-orbital separation in the quasi-one-dimensional Mott insulator Sr_2_CuO_3_. *Nature* **485**, 82–85 (2012). 22522933 10.1038/nature10974

[44] Dean, M. P. M. et al. Spin excitations in a single La_2_CuO_4_ layer. *Nat. Mater.* **11**, 850–854 (2012). 22941330 10.1038/nmat3409

[45] Squires, G. L. *Introduction to the Theory of Thermal Neutron Scattering* (Cambridge Univ. Press, 2012).

[46] Lines, M. E. Antiferromagnetism in a layer structure by green function techniques. *Phys. Rev.* **131**, 540–545 (1963).

[47] Lines, M. E. Magnetic properties of CoCl_2_ and NiCl_2_. *Phys. Rev.* **131**, 546–555 (1963).

[48] MacHado, T. & Dupuis, N. From local to critical fluctuations in lattice models: a nonperturbative renormalization-group approach. *Phys. Rev. E* **82**, 041128 (2010). 10.1103/PhysRevE.82.041128 21230259

[49] Zhang, L., Ren, J., Wang, J. S. & Li, B. Topological magnon insulator in insulating ferromagnet. *Phys. Rev. B* **87**, 144101 (2013).

[50] Mook, H. A. Temperature dependence of the spin dynamics of EuO. *Phys. Rev. Lett.* **46**, 508–511 (1981).

[51] Perring, T. G. et al. Spin waves throughout the Brillouin zone of a double-exchange ferromagnet. *Phys. Rev. Lett.* **77**, 711–714 (1996). 10062883 10.1103/PhysRevLett.77.711

[52] Dyson, F. J. General theory of spin-wave interactions. *Phys. Rev.* **102**, 1217–1230 (1956).

[53] Dyson, F. J. Thermodynamic behavior of an ideal ferromagnet. *Phys. Rev.* **102**, 1230–1244 (1956).

[54] Brookes, N. B. et al. Spin waves in metallic iron and nickel measured by soft X-ray resonant inelastic scattering. *Phys. Rev. B* **102**, 064412 (2020).

[55] Cooke, J. F., Lynn, J. W. & Davis, H. L. Calculations of the dynamic susceptibility of nickel and iron. *Phys. Rev. B* **21**, 4118–4131 (1980).

[56] Mook, H. A. & Nicklow, R. M. Neutron scattering investigation of the magnetic excitations in iron. *Phys. Rev. B* **7**, 336–342 (1973).

[57] Pelliciari, J. et al. Tuning spin excitations in magnetic films by confinement. *Nat. Mater.* **20**, 188–193 (2021). 33462465 10.1038/s41563-020-00878-0

[58] Korenman, V. & Prange, R. E. Anomalous damping of spin waves in magnetic metals. *Phys. Rev. B* **6**, 2769–2777 (1972).

[59] Xie, Y. et al. Spin excitations in metallic kagome lattice FeSn and CoSn. *Commun. Phys.* **4**, 240 (2021).

[60] Do, S. H. et al. Damped Dirac magnon in the metallic kagome antiferromagnet FeSn. *Phys. Rev. B* **105**, 180403 (2022).

[61] Liu, C. et al. Spin excitations and spin wave gap in the ferromagnetic Weyl semimetal Co_3_Sn_2_S_2_. *Sci. China Phys. Mech. Astron.* **64**, 217062 (2021).

[62] Zhang, Q. et al. Unusual exchange couplings and intermediate temperature Weyl state in Co_3_Sn_2_S_2_. *Phys. Rev. Lett.* **127**, 117201 (2021). 34558925 10.1103/PhysRevLett.127.117201

[63] Nag, A. et al. Correlation driven near-flat band Stoner excitations in a Kagome magnet. *Nat. Commun.* **13**, 7317 (2022). 36443343 10.1038/s41467-022-34933-y PMC9705307

