# Supplementary material for: Spin waves and orbital contribution to ferromagnetism in a topological metal
Source: Nat Commun. 2024 Oct 16;15:8905. doi: 10.1038/s41467-024-53152-1 (PMC11484860; doi:10.1038/s41467-024-53152-1)
Supplement: Supplementary file 1 — Supplementary Information [file 41467_2024_53152_MOESM1_ESM.pdf]

Supplementary Information for "Spin waves and orbital contribution to ferromagnetism in a topological metal"

Wenliang Zhang[1], Teguh Citra Asmara[1], Yi Tseng[1], Junbo Li[2], Yimin Xiong[3, 4], Yuan Wei[1], Tianlun Yu[1], Carlos William Galdino[1], Zhijia Zhang[1], Kurt Kummer[5], Vladimir N. Strocov[1], Y. Soh[1*], Thorsten Schmitt[1†], and Gabriel Aeppli[1, 6, 7, 8]

[1]*Paul Scherrer Institut, CH-5232 Villigen PSI, Switzerland*

[2]*Anhui Province Key Laboratory of Condensed Matter Physics at Extreme Conditions, High Magnetic Field Laboratory, Chinese Academy of Sciences, Hefei 230031, China*

[3]*Department of Physics, School of Physics and Optoelectronics Engineering, Anhui University, Hefei 230601, P. R. China*

[4]*Hefei National Laboratory, Hefei 230028, China*

[5]*European Synchrotron Radiation Facility, 71 Avenue des Martyrs, Grenoble F-38043, France*

[6]*Department of Physics, ETH Zurich, CH-8093 Zurich, Switzerland.*

[7]*Quantum Center, ETH Zurich, CH-8093 Zurich, Switzerland.*

[8]*Institut de Physique, EPF Lausanne, CH-1015 Lausanne, Switzerland.*

To whom correspondence should be addressed: *yona.soh@psi.ch; †thorsten.schmitt@psi.ch

Supplementary Note 1: X-ray absorption

The X-ray absorption spectra (XAS) are measured in the total electron yield (TEY) mode by recording the drain current from the sample. The relation between TEY and the absorption coefficient μ in a bulk sample can be described by[1–3]:

$$\mathrm{TEY}(E_\mathrm{i},\alpha) = M\frac{\mu(E_\mathrm{i},\,\boldsymbol{\varepsilon})l_\mathrm{e}}{\sin\alpha}\cdot\frac{1}{1+\mu(E_\mathrm{i},\,\boldsymbol{\varepsilon})l_\mathrm{e}/\sin\alpha} \qquad \text{(Supplementary Equation 1)}$$

where M is a constant dependent on the material and the intensity of the incident photons, E_i is the incident photon energy, $\boldsymbol{\varepsilon}$ is the photon polarization, l_e is the electron escape depth, and α is the incident angle. When $\mu l_\mathrm{e}/\sin\alpha \ll 1$ (l_e is much smaller than the photon penetration depth $l_\mathrm{p} = \sin\alpha/\mu$), TEY is simply proportional to μ. This is usually true in transition metals around the L edge with not too small α. However, this condition is not fully met when E_i is close to the absorption edge (where μ becomes large) and α is small (grazing incidence), which will reduce the signal of TEY and thus distort the proportionality between TEY and μ as E_i is varied, known as the saturation effect. The exact μ is determined by the measured TEY as:

$$\frac{\mu(E_\mathrm{i},\,\boldsymbol{\varepsilon})l_\mathrm{e}}{\sin\alpha} = \frac{\mathrm{TEY}(E_\mathrm{i},\alpha)/M}{1-\mathrm{TEY}(E_\mathrm{i},\alpha)/M} \qquad \text{(Supplementary Equation 2)}$$

We first measured the XAS with linear vertical (σ) polarization at several different α's to estimate M, see Supplementary Figure 1a. Supplementary Figure 1b shows the angle dependence of the TEY $*\sin\alpha$ at the pre-edge (blue circles, E_i = [704, 705] eV) and resonance (red squares, E_i = [707.3, 707.9] eV), and the solid lines are fitted by $M\cdot r/(1+r/\sin\alpha)$ (see

Supplementary Equation 1), where $r=\mu l_e$ and M are both varied. Supplementary Figure 1c shows the fitted M at different E_i. Here, we take the weighted average as the value of M, where the weights are the amplitudes of TEY at normal incidence. Supplementary Figure 1d shows the measured XAS around the Fe L_3 edge for circular polarizations at different angles. Using Supplementary Equation 2 and the value of M, we then obtain new estimates for μl_e. The results are plotted in Supplementary Figure 1e, with the error bars representing the change of the curve shape caused by the deviation from the average M value by a standard error.

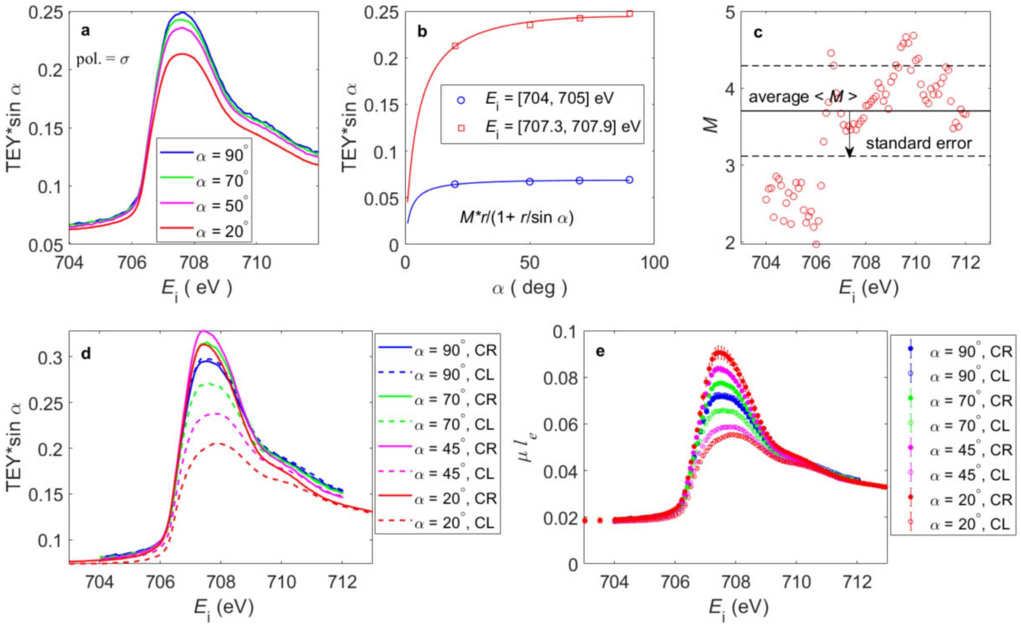

Supplementary Figure 1. (**a**) XAS measured by TEY with σ polarization at different incident angle α. (**b**) Incident angle dependence of TEY $\cdot \sin\alpha$ at pre-edge energy (averaged in E_i = [704, 705] eV, blue circle) and at resonance energy (averaged in E_i = [707.3, 707.9] eV, red square); the solid lines are fitted by $M \cdot r/(1 + r/\sin\alpha)$. (**c**) The fitted Mvalues as a function of incident energy; the solid black line indicates the weighted average value, and the dashed lines indicate a standard error deviation. (**d**) The measured XAS with circular polarizations at different incident angles. (**e**) The corrected μl_e according to Supplementary Equation 2. The measurements were done at 25 K.

Supplementary Note 2: Self-absorption correction of the RIXS spectra over the whole energy range

The self-absorption effect in RIXS spectra is corrected according to[4,5]:

$$I_{\text{exp}}(E, \boldsymbol{\varepsilon}_i) = \sum_{\boldsymbol{\varepsilon}_o} \frac{I_c(E, \boldsymbol{\varepsilon}_i, \boldsymbol{\varepsilon}_o)}{\mu(E_i, \boldsymbol{\varepsilon}_i, \mathbf{k}_i) + \mu(E_o, \boldsymbol{\varepsilon}_o, \mathbf{k}_o) \cdot \sin\alpha / \sin\theta} \quad \text{(Supplementary Equation 3)}$$

Here, α and θ are the incident and outgoing angles, respectively (Figure 1c), $E = E_i - E_o$ is the energy transfer, and I_c is the intrinsic RIXS cross section without self-absorption. Since the outgoing polarization ($\boldsymbol{\varepsilon}_o$) is not resolved, the experimental result (I_{exp}) sums all $\boldsymbol{\varepsilon}_o$. The absorption coefficient and its angle dependence are determined by the XAS as described in Supplementary Note 1 above. Supplementary Figure 2 shows the angle dependence of the

denominator in Supplementary Equation 3 for CL and CR outgoing polarization and their ratio at $E = 0.1$ eV. The difference between the two outgoing polarizations is relatively small in the studied momentum range. For even higher energy, the difference in μ with different polarizations becomes even smaller. It is therefore a good approximation to use the average $\mu(E_\mathrm{o}, \boldsymbol{\varepsilon}_\mathrm{o}, \mathbf{k}_\mathrm{o})$ of the two outgoing polarizations in the denominator. The summation symbol then only applies to the numerator, and we can get the intrinsic RIXS cross-section $\sum_{\boldsymbol{\varepsilon}_\mathrm{o}} I_\mathrm{c}\,(E, \boldsymbol{\varepsilon}_\mathrm{i}, \boldsymbol{\varepsilon}_\mathrm{o})$ by simply multiplying the experimental RIXS spectra I_exp with the denominator. The spectra corrected in this way are shown in Fig. 3 in the main text. We note that the spectra with highest in-plane momentum **q** = (0.39, 0, 1.17) show obviously strong elastic peaks due to the grazing incident angle (α = 10°) where the X-rays partially illuminate the sample edges with rough surfaces and glue. Such elastic scattering was not affected by the self-absorption effect. Therefore, to avoid inducing artificial dichroism on elastic peaks in CR and CL polarizations, we keep the two elastic peaks having same correction: the elastic peak in CL spectrum here is first fitted by the resolution function and then corrected by the self-absorption factor of CR incidence, while the rest of the spectral weight is still corrected by the self-absorption factor of CL incidence.

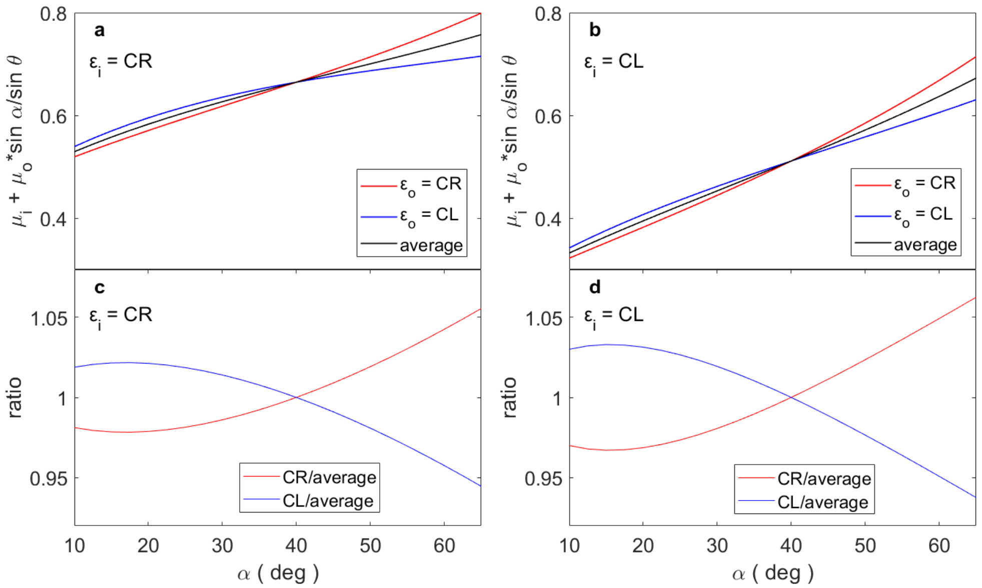

Supplementary Figure 2. The angle dependence of the self-absorption factor (denominator in Supplementary Equation 3) at $E = 0.1$ eV. (**a**) The angle dependence for CR (red) and CL (blue) outgoing polarizations and their average (black) with CR incidence polarization. (**b**) The same as (a) but with CL incident polarization. (**c**) shows the ratio of the self-absorption factor of CR (red) and CL (blue) outgoing polarization to the average self-absorption factor for CR incidence beam. (**d**) the same as (**c**) but for CL incident beam.

Supplementary Note 3: Polarization factors in RIXS cross-section for spin-wave excitations

As shown in the Methods of the main text, the RIXS cross-section for spin wave excitations is described by equation (5) with polarization factors $P_a^* P_b$. In the scattering geometry as in Fig. 1b of the main text, we define the polarization vectors for incident and outgoing X-rays as:

$$\boldsymbol{\varepsilon}_{\mathbf{i,L}} = \frac{-i\sin\alpha\,, 1, -i\cos\alpha}{\sqrt{2}}$$

$$\boldsymbol{\varepsilon}_{\mathbf{i,R}} = \frac{i\sin\alpha\,, 1, i\cos\alpha}{\sqrt{2}}$$

$$\boldsymbol{\varepsilon}_{\mathbf{o,L}} = \frac{i\sin(\alpha+\beta)\,, 1, i\cos(\alpha+\beta)}{\sqrt{2}}$$

$$\boldsymbol{\varepsilon}_{\mathbf{o,R}} = \frac{-i\sin\,(\,\alpha+\beta)\,, 1, -i\cos(\alpha+\beta)}{\sqrt{2}}$$

(Supplementary Equation 4)

Here L and R indicate CL and CR polarizations, respectively. For ferromagnets with moments along the x direction, we only consider the transverse excitations, *i.e.*, S^{yy}, S^{zz}, S^{yz}, and S^{zy}. The RIXS cross-sections for different incident and outgoing circular polarizations can be written as:

$$\begin{aligned} I_{c_\mathrm{i},c_\mathrm{o}} = {} & (\sin\beta)^2 \cdot S^{yy}(\boldsymbol{q},\omega) + [c_\mathrm{o}\cdot\sin\alpha - c_\mathrm{i}\cdot\sin(\alpha+\beta)]^2 \cdot S^{zz}(\boldsymbol{q},\omega) \\ & +[c_\mathrm{o}\cdot\sin\alpha - c_\mathrm{i}\cdot\sin(\alpha+\beta)]\cdot\sin\beta\cdot iS^{yz}(\boldsymbol{q},\omega) \\ & -[c_\mathrm{o}\cdot\sin\alpha - c_\mathrm{i}\cdot\sin(\alpha+\beta)]\cdot\sin\beta\cdot iS^{zy}(\boldsymbol{q},\omega) \end{aligned}$$

(Supplementary Equation 5)

Here $c_{\mathrm{i(o)}}$ = +1 or -1 for CL or CR polarizations, respectively.

Supplementary Note 4: Angular dependence of the RIXS MCD intensity of the low-energy excitations

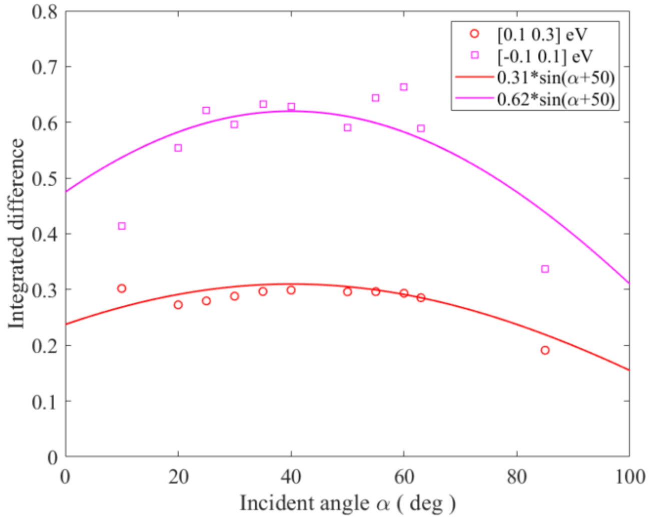

Supplementary Figure 3. Integrated intensities of RIXS MCD (E_i = 707.4 eV) at T = 25 K in Figure 3**b** of the main text, in an energy interval [0.1 eV, 0.3 eV] (red circles) and [-0.1 eV, 0.1 eV] (magenta squares) as a function of incident angle α, which correspond to the flat mode and acoustic mode, respectively. The solid lines indicate the $\sin(\alpha + 50°)$ curves.

Supplementary Note 5: Correcting low-energy RIXS MCD for spin-wave excitations by self-absorption and polarization factors

The diffuse reflection from the sample surface, which contributes to the elastic scattering at zero-energy loss, is neither affected by the self-absorption effect nor the different circular polarizations. To completely subtract its contributions and obtain the correct profiles of the low-energy spin excitations, we first perform the direct subtraction between the measured RIXS spectra of CR and CL polarizations without doing any corrections in advance. The obtained differences (RIXS MCD) contain the pure signals from the sample but are still influenced by both self-absorption and polarization factors. As the absorption coefficients and polarization factors of spin-wave excitations are all known, we can then exactly correct the low-energy RIXS MCD and obtain the profiles for the spin-spin correlations $S(\mathbf{q},\omega)$. Below we show how the corrections are done for the low-energy RIXS MCD. For ferromagnetic spin waves in our Fe_3Sn_2 sample, $S^{yy}=S^{zz}=iS^{yz}=-iS^{zy}\equiv S(\mathbf{q},\omega)$. According to Supplementary Equation 5, we have a total polarization factor defined by the scattering angles:

$$p(\boldsymbol{\varepsilon}_\mathrm{i},\boldsymbol{\varepsilon}_\mathrm{o},\alpha,\beta)=(\sin\beta)^2+[c_\mathrm{o}\cdot\sin\alpha-c_\mathrm{i}\cdot\sin(\alpha+\beta)]^2$$
$$+2[c_\mathrm{o}\cdot\sin\alpha-c_\mathrm{i}\cdot\sin(\alpha+\beta)]\cdot\sin\beta$$

(Supplementary Equation 6)

The self-absorption correction factor for distinct incident and outgoing polarizations is:

$$s_a(E_\mathrm{i},E_\mathrm{o},\boldsymbol{\varepsilon}_\mathrm{i},\boldsymbol{\varepsilon}_\mathrm{o},\alpha,\theta)=\frac{1}{\mu(E_\mathrm{i},\boldsymbol{\varepsilon}_\mathrm{i},\mathbf{k}_\mathrm{i})+\mu(E_\mathrm{o},\boldsymbol{\varepsilon}_\mathrm{o},\mathbf{k}_\mathrm{o})\cdot sin\,\alpha/sin\,\theta}$$

(Supplementary Equation 7)

With the two factors, we obtain the relation between the direct RIXS MCD cross-sections and the spin-spin correlations of spin waves:

$$I_{\mathrm{direct-MCD}}=\left[\sum_{\varepsilon_o}p(\boldsymbol{\varepsilon}_\mathrm{i},\boldsymbol{\varepsilon}_\mathrm{o})\cdot s_a(\boldsymbol{\varepsilon}_\mathrm{i},\boldsymbol{\varepsilon}_\mathrm{o})-p(\boldsymbol{\varepsilon}_\mathrm{i}',\boldsymbol{\varepsilon}_\mathrm{o})\cdot s_a(\boldsymbol{\varepsilon}_\mathrm{i}',\boldsymbol{\varepsilon}_\mathrm{o})\right]\cdot S(\mathbf{q},\omega)$$

(Supplementary Equation 8)

Supplementary Note 6: Out-of-plane momentum (*L*) dependence of the spin-wave excitations

Supplementary Figure 4 and 5 show the out-of-plane momentum (*L*) dependence of the RIXS spectra collected at ESRF and SLS, respectively. At the ESRF, we measured from $L = 2.1$ to $L = 1.1$ in sequence. The sample surface degraded with time, as can be seen from the suddenly increased elastic peak intensity at $L = 1.1$, which was measured ~ 12 hours after the sample was cleaved. The degradation is due to the initial cleaving in a vacuum of ~1×10^{-8} mbar at room temperature (see Method in the main text) and slightly worse vacuum in the main chamber for the measurements at ID32 of ESRF (~1×10^{-9} mbar), compared to the other measurements done at ADRESS of SLS (~2×10^{-10} mbar). The solid lines in Supplementary Figure 4c are the fittings to two damped harmonic oscillators convolved with a Gaussian resolution function with FWHM ~35 meV. The solid lines in Supplementary Figure 5c and 5d

are the fittings to two damped harmonic oscillators convolved with a resolution function with FWHM ~80 meV (L = 1.34, 1.5, 1.77) and 74 meV (L = 1.85, 1.97).

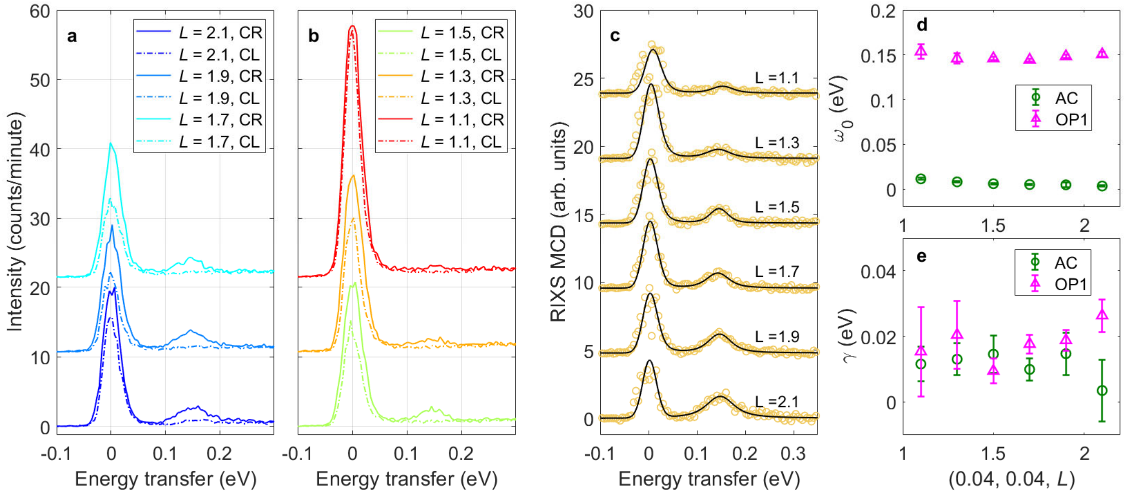

Supplementary Figure 4. Out-of-plane momentum (*L*) dependence of the low-energy RIXS spectra collected at ESRF. (**a**) and (**b**) RIXS spectra with CR and CL incident X-rays. The in-plane momentum is fixed at $\mathbf{q}_{//}$ = (0.04, 0.04). (**c**) RIXS MCD (circles) and the fitting to two damped harmonic oscillators (solid lines). The results are already corrected by polarization and self-absorption factors as described above. (**d**) and (**e**) are the fitted bare frequencies and damping factors, respectively. The error bars are the standard errors of fitted parameters. The measurements were done at 25 K with incident energy at Fe L_3 resonance.

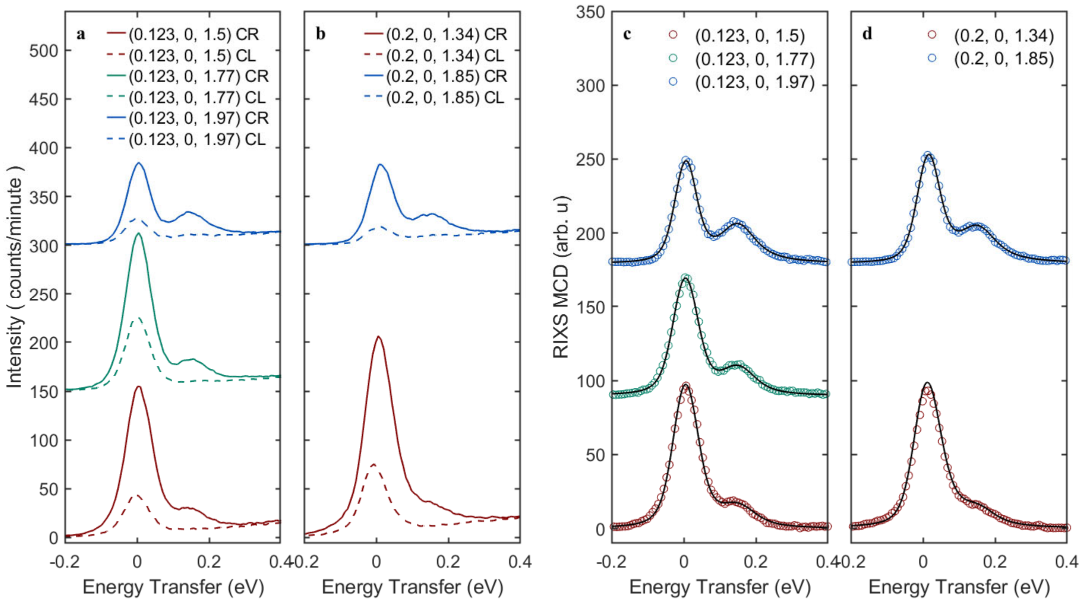

Supplementary Figure 5. Out-of-plane momentum (*L*) dependence of the low-energy RIXS spectra collected at SLS. (**a**) and (**b**) RIXS spectra with CR and CL incident X-rays. The in-plane momentum is fixed at $\mathbf{q}_{//}$ = (0.123, 0) and $\mathbf{q}_{//}$ = (0.2, 0), respectively. (**c**) and (**d**) RIXS

MCD (circles) and the fitting to two damped harmonic oscillators (solid lines). The results are already corrected by polarization and self-absorption factors as described above.

Supplementary Note 7: Fitting of the spin-wave excitations

Supplementary Figure 6 displays the fitting results of the low-energy RIXS MCD to the J_1-J_{bi} Heisenberg model with damped harmonic oscillator profiles, which have a form:

$$S(\omega) = (n(\omega) + 1) \cdot \frac{\gamma\omega}{\left(\omega^2 - \omega_0^2\right)^2 + 4\gamma^2\omega^2} \qquad \text{(Supplementary Equation 9)}$$

where $n(\omega) + 1$ is the Bose factor. The fitting energy range is [-0.35 0.35] eV to avoid the influence from the tails of the high-energy fluorescence. The damped harmonic oscillators are convolved with a resolution function of Voigt profile with FWHM = 74 meV, Gaussian standard deviation σ=24.3, Lorentzian HWHM γ=13.7; the parameters are determined by fitting the elastic peak measured on a carbon tape at the sample position. The fitting parameters are shown in the main text and Fig. 5.

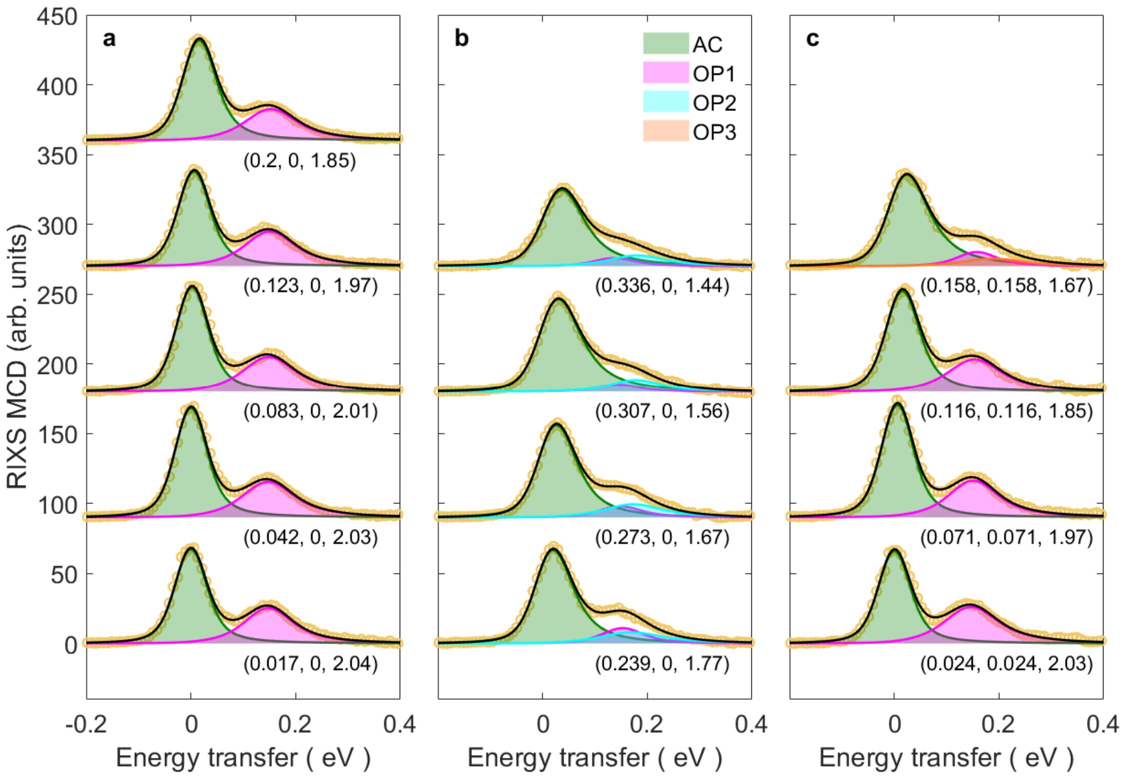

Supplementary Figure 6. The low-energy RIXS-MCD spectra (yellow circles) and the fitting curves to J_1-J_{bi} Heisenberg model. (**a-c**) show the spectra at different momenta as labelled in the figure. The green, magenta, cyan, and orange curves and patches represent the fitted AC, OP1, OP2, and OP3 modes shown in Fig 1**d** of the main text, respectively.

Comparison to Fano line-shape fitting

In metallic materials, the interference from the continuum of the electron hole pair excitations can modify the observed collective excitations when they appear at the same energy scale. The Fano line-shape describes the interference between the excitations from a discrete state and a

continuum[6], and thus can be an appropriate line-shape for the collective excitations in metallic materials. Here we also test the fitting to a Fano line-shape:

$$I(\omega) = \frac{\left(q_f \cdot \gamma + (\omega - \omega_0)\right)^2}{\gamma^2 + (\omega - \omega_0)^2} \qquad \text{(Supplementary Equation 10)}$$

Here γ describes the linewidth, and q_f is the Fano parameter, which indicates the ratio of resonant scattering to the background scattering amplitude. When q_f*γ is very large compared to ω_0, the line-shape becomes a Lorentzian, which means the interference from the continuum is weak. In the fitting, we set the contribution on the negative energy (energy gain) side of the line-shape to be zero and convolute it with the resolution function. Supplementary Figure 7 shows the fitting results. While the fitting gives qualitatively similar results as the DHO fitting, it is overall worse especially at large $\mathbf{q}_{//}$ as indicated by the large error bars in the damping but also at small $\mathbf{q}_{//}$ shown by the deviations around the pre-peak positions at negative energy transfer. Supplementary Figure 7f shows the values of q_f*γ/ω_{max}, which are mostly very large and suggest the fitting profiles are close to Lorentzian.

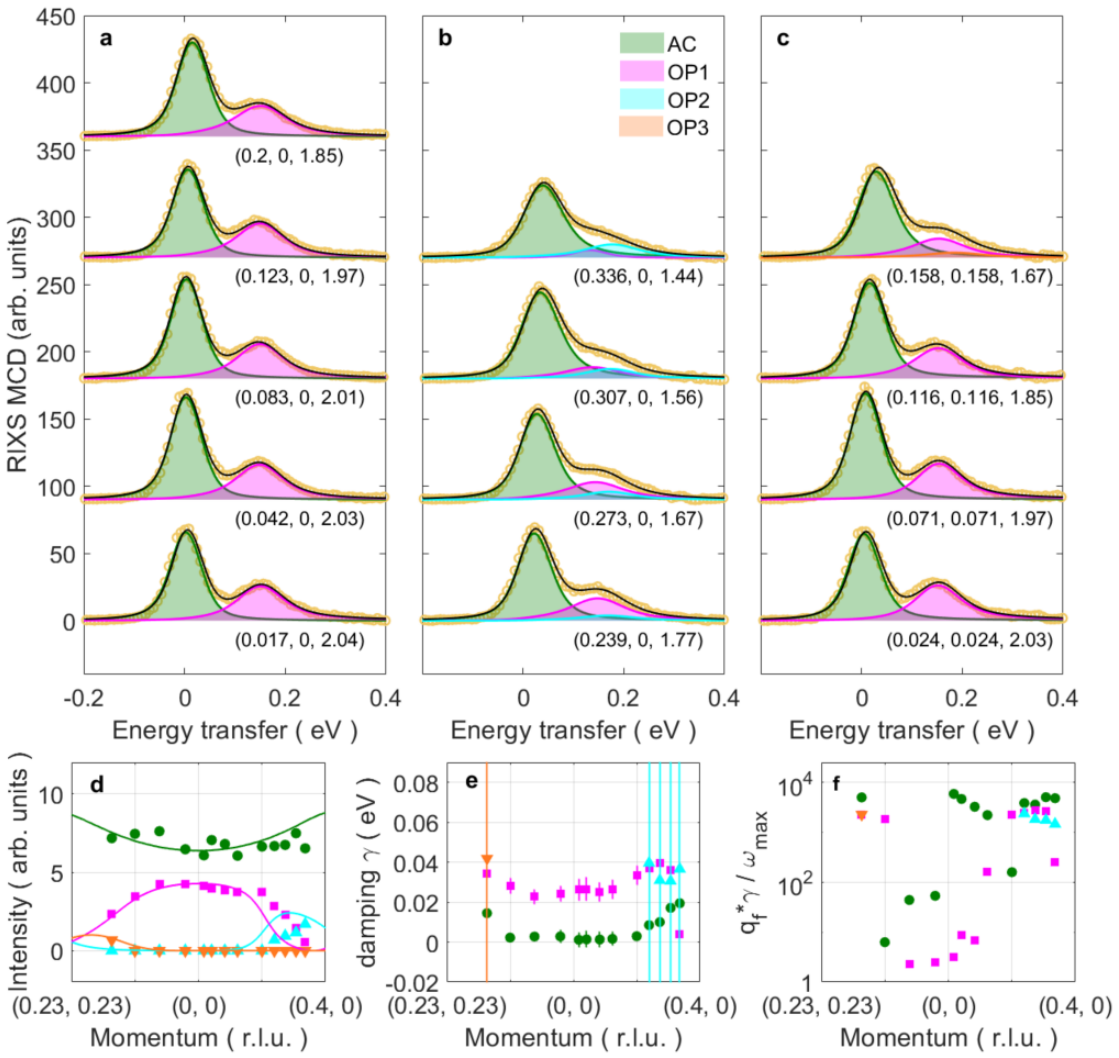

Supplementary Figure 7. Fano line-shape fitting to J_1-J_{bi} Heisenberg model. (**a-c**) The RIXS MCD and the fitting curves. (**d**) The integrated intensities (peak areas) of the fitted acoustic mode (green circles) and the optical modes (magenta (OP1), cyan (OP2), orange (OP3)). (**e**) The fitted damping factors γ. (**f**) Fano parameter q_f multiplied by γ/ω_{max}. The error bars are the standard errors of fitted parameters.

Supplementary Note 8: Effects of other magnetic interactions

Here we consider the effects of additional magnetic interactions discussed in the main text, such as the inter-bilayer interaction J_c, and the difference between J_{1a} and J_{1b}, on the spin waves. The linear spin wave dispersion and correlations are simulated based on the SpinW package. Supplementary Figure 8 shows the results of including an inter-bilayer interaction $J_c = -8$ meV in the J_1-J_{bi} model, with $J_1 = -25.0$ meV and $J_{bi} = -37.5$ meV as in the main text. Supplementary Figure 9 shows the results of introducing an 8 meV difference between J_{1a} and J_{1b} in the J_1-J_{bi} model. Supplementary Figure 10 shows the results of including an in-plane next-nearest-neighbour interaction $J_2 = \pm 0.15\ J_1$ in the J_1-J_{bi} model.

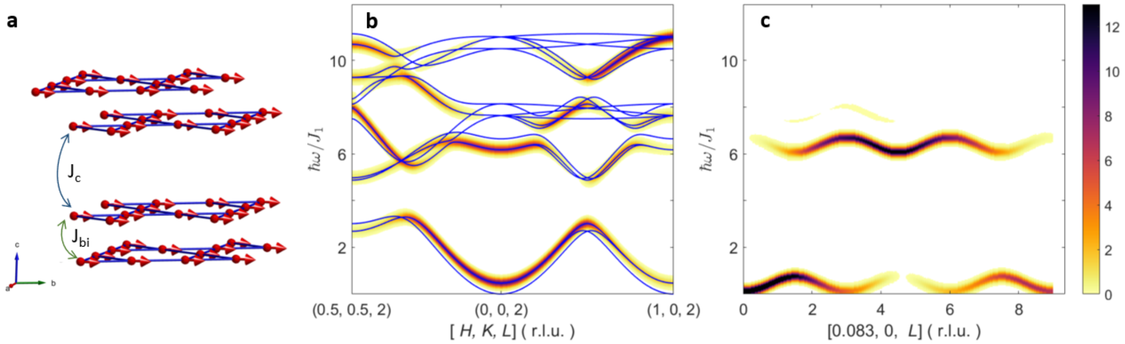

Supplementary Figure 8. Spin wave dispersion and the spin-spin correlations with inter-bilayer interaction $J_c = -8$ meV added in the J_1-J_{bi} model. (**a**) Lattice structure and illustration of J_{bi} and J_c interactions. The Sn atoms are not shown. (**b**) Dispersion and spin-spin correlations $Im(S^{zy}(\boldsymbol{q},\omega) - S^{yz}(\boldsymbol{q},\omega))$ broadened by a Gaussian with FWHM = 7 meV. (**c**) Out-of-plane momentum dependence of the spin-spin correlations at $\mathbf{q}_{//} = (0.083, 0)$.

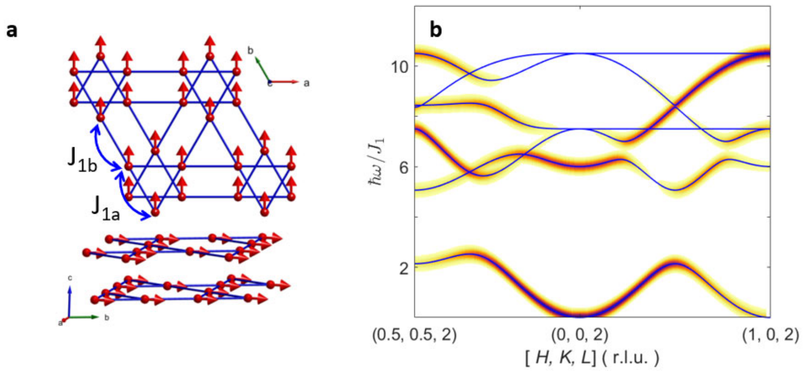

Supplementary Figure 9. Spin wave dispersion and the spin-spin correlations with different J_{1a} and J_{1b} in the J_1-J_{bi} model; here J_{1a} = -29 meV and J_{1b} = -21 meV. (**a**) Lattice structure and illustration of J_{1a} and J_{1b} interactions in the breathing triangles. (**b**) Dispersion and spin-spin correlations $Im(S^{zy}(\boldsymbol{q},\omega) - S^{yz}(\boldsymbol{q},\omega))$ broadened by a Gaussian with FWHM = 7 meV.

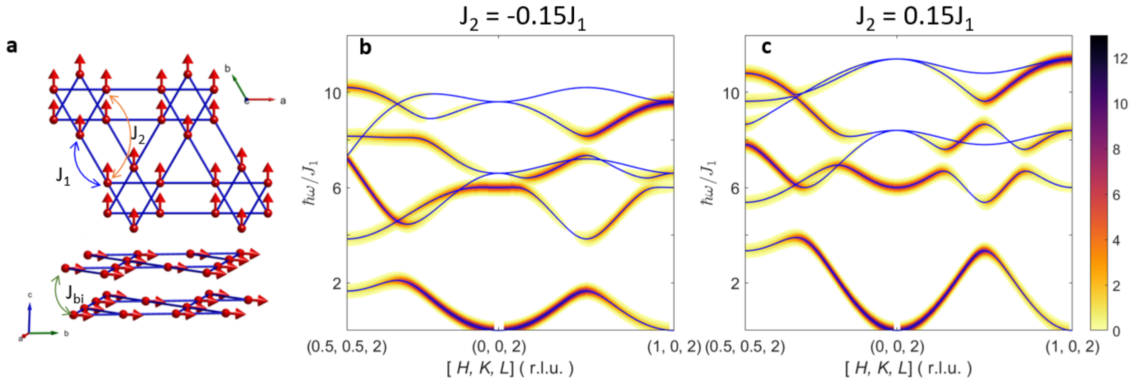

Supplementary Figure 10. Spin wave dispersion and the spin-spin correlations with interaction $J_2 = \pm 0.15\, J_1$ added into the J_1-J_{bi} model with J_1 = -25 meV and J_{bi} = -37.5meV. (**a**) Lattice structure and illustration of J_1, J_{bi}, and J_2 interactions. (**b**) and (**c**) Dispersion and spin-spin correlations $Im(S^{zy}(\boldsymbol{q},\omega) - S^{yz}(\boldsymbol{q},\omega))$ broadened by a Gaussian with FWHM = 7 meV, for J_2 = -0.15 J_1 and J_2 = 0.15 J_1, respectively.

Supplementary References

1. Nakajima, R., Stöhr, J. & Idzerda, Y. U. Electron-yield saturation effects in L-edge X-ray magnetic circular dichroism spectra of Fe, Co, and Ni. *Phys. Rev. B* **59**, 6421–6429 (1999).

2. Henneken, H., Scholze, F. & Ulm, G. Lack of proportionality of total electron yield and soft X-Ray absorption coefficient. *J. Appl. Phys.* **87**, 257–268 (2000).

3. Ruosi, A. *et al.* Electron sampling depth and saturation effects in perovskite films investigated by soft X-Ray absorption spectroscopy. *Phys. Rev. B* **90**, 125120 (2014).

4. Chabot-Couture, G. *et al.* Polarization dependence and symmetry analysis in indirect K -edge RIXS. *Phys. Rev. B* **82**, 035113 (2010).

5. Achkar, A. J. *et al.* Bulk sensitive X-Ray absorption spectroscopy free of self-absorption effects. *Phys. Rev. B* **83**, 81106(R) (2011).

6. Fano, U. Effects of configuration interaction on intensities and phase shifts. *Phys. Rev.* **124**, 1866–1878 (1961).
